# Molecular Models for the Core Components of the Flagellar Type-III Secretion Complex

**DOI:** 10.1371/journal.pone.0164047

**Published:** 2016-11-17

**Authors:** William R. Taylor, Teige R. S. Matthews-Palmer, Morgan Beeby

**Affiliations:** 1 Laboratory of Computational Cell and Molecular Biology, Francis Crick Institute, 1 Midland Rd., London NW1 1AT, United Kingdom; 2 Department of Life Sciences, Imperial College, London, United Kingdom; Nanyang Technological University, SINGAPORE

## Abstract

We show that by using a combination of computational methods, consistent three-dimensional molecular models can be proposed for the core proteins of the type-III secretion system. We employed a variety of approaches to reconcile disparate, and sometimes inconsistent, data sources into a coherent picture that for most of the proteins indicated a unique solution to the constraints. The range of difficulty spanned from the trivial (FliQ) to the difficult (FlhA and FliP). The uncertainties encountered with FlhA were largely the result of the greater number of helix packing possibilities allowed in a large protein, however, for FliP, there remains an uncertainty in how to reconcile the large displacement predicted between its two main helical hairpins and their ability to sit together happily across the bacterial membrane. As there is still no high resolution structural information on any of these proteins, we hope our predicted models may be of some use in aiding the interpretation of electron microscope images and in rationalising mutation data and experiments.

## Introduction

Combating pathogens would benefit from mechanistic insights into the molecular machinery involved in infection. Bacterial type III secretion systems (T3SSs) are inner membrane complexes that guide protein self-assembly to form large periplasmic structures. T3SSs are responsible for the assembly of two closely related, determinate structures: flagella, which are propellers used for bacterial motility; and injectisomes, which are molecular syringes that use their T3SS to inject effector proteins in to host cells.

A transmembrane export gate comprising five proteins forms the mechanistic core of the T3SS. The export gate has an essential role in energizing secretion as well as coordinating substrate-specificity for ordered assembly. The five export gate proteins are FlhA, FlhB, FliP, FliQ and FliR under flagella nomenclature (SctV, SctU, SctR, SctS, SctT in unified injectisome nomenclature), and are conserved across flagella and injectisomes. In contrast to the non-essential cytoplasmic ATPase complex [1–3], the export gate proteins are all essential for secretion [4]. The export gate harnesses a proton motive force [3] to drive substrate proteins out through a 2nm channel in the filamentous assembly [5], requiring substrates to be unfolded for secretion [6]. A structural protein of the FliF family forms a confining ring (MS ring) of 24nm inner diameter [7] in the inner membrane around the export gate [8, 9]. It is unknown whether a lipid bilayer exists within the MS ring, but all five export gate proteins are strongly predicted to be alpha-helical transmembrane proteins from their sequences. The cytoplasmic domain of FlhA is known to form a nonameric ring [10] and to bind the chaperones of late substrates, which has been suggested to finely control the assembly of the pentameric cap on the growing flagellum [11]. The transmembrane domain of FlhA may form a proton pore for transduction of the PMF [3, 12]. FlhB cytoplasmic domain participates with a “molecular ruler” to switch substrate specificity when flagellar hooks and injectisome needles reach their full length [13].

Insight into the molecular mechanism of T3S has been hampered by the lack of information on the structure of this five-protein export gate. Most work has centered upon the cytoplasmic regions, with structures of flagellar C-ring [14–16] and ATPase complex proteins [17, 18] obtained by X-ray crystallography and located *in situ* by electron cryo-microscopy methods [10, 19–21]. Considerably less is known about the structures of the transmembrane proteins, due partly to the intrinsic challenges these proteins pose to crystallograpic and electron cryo-microscopic methods. Whilst structural information has been obtained for a FliF homolog [22] and the cytoplasmic domains of FlhA [11, 23] and FlhB [24] homologs, the structures of the transmembrane domains of the five export gate proteins remain completely unknown, despite these being the most critical mechanistic proteins.

The recent success of correlated substitution analysis in the prediction of globular protein structure has also been extended to the more constrained case of *α*-helical transmembrane proteins, where its success has been striking [25]. (See [26], for a review). Correlated substitution analysis is used to generate distance constraints for model construction, under the assumption that if mutation at one residue is correlated with mutation at another residue, then they are likely to be proximal in the protein fold. The success of these methods depends on having typically thousands of sequences in a multiple sequence alignment. Since the export gate proteins are sufficiently widespread in bacteria, we investigated the application of correlated substitution methods to predict their possible structures.

Here we describe a novel consensus approach to the analysis of correlated substitution results; combining the predicted contacts from three different methods and using these to construct molecular models generated by up to four different modelling approaches.

## Materials and Methods

The recent success of correlated substitution analysis in the prediction of globular protein structure has also been extended to transmembrane (TM) protein structure in which the TM segments are alpha helical [26]. Results for this class of protein have been even more successful due to their more limited conformational variation, being composed essentially of just one type of (alpha) secondary structure with helices that are grenerally larger than those found in globular proteins and are predominantly aligned in a single direction so they can cross the lipid membrane [25, 27]. However, the success of all these methods, applied to both globular and TM proteins, depends on having typically thousands of sequences in a multiple sequence alignment. With less than 1000 sequences in the alignment, the predicted contacts are generally not accurate enough to define a unique structure. Fortunately, there are currently sufficient bacterial genomes that any bacterial protein family that is reasonably widespread can expect easily to exceed the 1000 sequence threshold criterion. As the proteins of the type-III secretion system (including the flagellar associated members) are indeed widespread, we investigated the application of correlated substitution methods to predict their possible structures.

Given that the fundamental limitation of sufficient sequences is met, there follow choices to be made in how these are filtered or weighted to remove or down-weight close homologues, and how gaps are treated. There is then a choice of methods that can be used to calculate co-varying sites and for each of these, a cutoff or weight can be applied to quantify which predicted contacts can be trusted. Given a set of likely contacts, the construction of a protein model to best account for these is not a simple or well defined problem and different methods and also repeated runs of the same method will produce different structures that cannot easily be distinguished within the uncertainty associated with the predicted contacts. In addition to these choices that are intrinsic to the approach, there is also choices to be made on the degree to which other extrinsic constraints should be applied, such as the statistical prediction of TM-helical segments (also by a variety of methods), the prediction of lipid exposure and any external cross-linking constraints.

**Fig 1:**
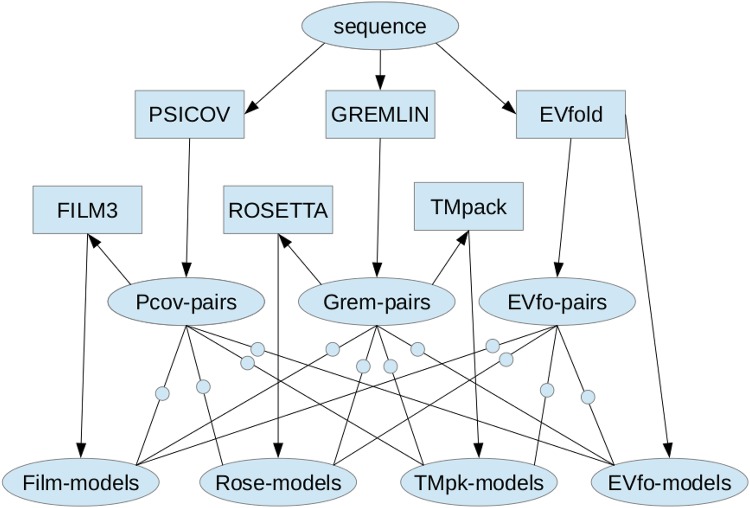
Overview of the Method. A protein **sequence** is presented to the programs **PSICOV**, **GREMLIN** and **EVfold**, each of which construct a multiple sequence alignment that is analysed for covariation between columns, resulting in ranked list of predicted contacts (**X-pairs**) for each method (**X**). Using these pairs, models are constructed, automatically by the EVfold server and in separate applications using the methods **FILM3**, **ROSETTA** and **TMpack**. Each set of models is then scored against each set of contacts (small **circles**) using a common evaluation function and the models ranked. The top 10 models from each set of contacts (whatever their construction method) were then combined into a consensus structure using the program MODELLER.

Our approach in this work is to use all available methods to generate both constraints and models and combine these to produce a final consensus model. Fig 1 gives an overview of the methods that we used, all of which will be described below.

### Coevolution analysis

Each of the co-evolution analysis methods described below also perform their own sequence search using either jackHmmer or a similar program. In each server we accepted the default search parameters.

**PSICOV:** Sequences were submitted to the PSICOV server at: http://bioinf.cs.ucl.ac.uk/MetaPSICOV/ and also calculated using a local (in-house) copy if the original program [28].

**Gremlin:** Sequences were submitted to the Gremlin server at: http://gremlin.bakerlab.org/submit.php [29].

**EVfold:** Sequences were submitted to the EVfold server at: http://evfold.org/evfold-web/evfold.do [30].

**Contact segment parsing:** Following a long tradition on the analysis of packing of *α*-helices in globular proteins based on the analysis of hydrophobic stripes [31–35], a similar approach was adopted to the analysis of features in the predicted contact maps. These often exhibit stripes parallel to or orthogonal to the contact map diagonal indicating parallel and antiparallel packing respectively. These trends were automatically assigned according to the relative strengths of a regression line fitted in both directions with the additional refinement that the total fit over all helices was maximised by allowing the fitted segment ends to shift. A solution to this problem was obtained using dynamic programming [36], similar to the applications to transmembrane segment prediction [38] and parsing linear structural segments [39]. Given the parallel/antiparallel scores for each interaction, it would be possible to select the best overall assignment of orientations that are consistent with an alternating in/out topology. However, we did not impose this as it has the danger of obscuring missing helices (that were not predicted) or re-entrant helices. The consistency of the unbiased local assignments also gives some indication of the quality of the predicted contacts.

### Model construction

**TM-helix prediction:** Sequences were extracted from the non-redundant NCBI sequence databank using the jackHmmer program [40] and reduced to a small non-redundant selection for sequence analysis [41] which included secondary structure prediction using PsiPred ([42]). TM helices were predicted as a consensus of the methods caluclated by the TOPCONS server (http://topcons.cbr.su.se/) [43], supplemented by the MEMSAT methods [38, 44, 45]. Although individual prediction piplines associated with each model construction method apply their own (ususlly in-house) method, for consistencey in comparison, we preferred to use an external method that was common to all and so focused on the TOPCONS consensus.

**Fig 2:**
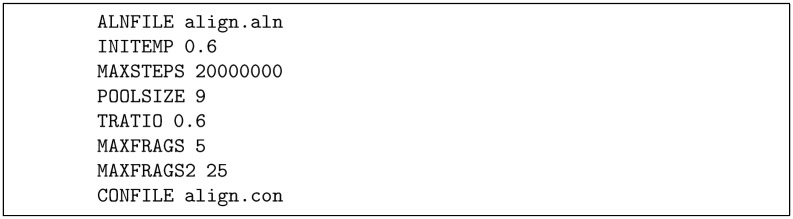
FILM3 runfile for a run-length of 20 million steps.

**FILM3:** The program FILM3 [25] is a specialised version of the MEMPACK program [46, 47] incorporating correlated mutation constraints. These programs follow the fragment assembly approach of the FRAGFOLD program but adapted for TM-proteins to include a pre-calculation of the TM-segments and an optional constraint that can be applied to their residue positions relative to the membrane (Z-filter). Otherwise the program uses a Monte-Carlo (MC) approach to assemble fragments of protein structure to satisfy the given constraints. By its nature, MC modelling is a rather haphazard process and requires many attempts over many iterations to obtain successful (compact) solutions. For each condition tested we therefore made typically 100 models trying from 10 up to 50 million iterations. Tests were carried out both using the PSICOV, EVfold and GREMLIN constraints. As discussed further below, the program was applied without the Z-filter constraint. A parameter file for a 20M step run is shown in Fig 2.

**Fig 3:**
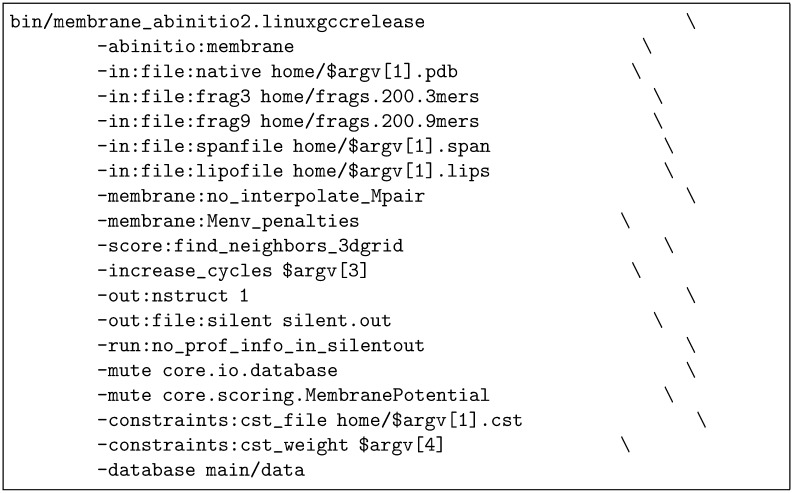
ROSETTA runfile as a shell script for which the arguments were: 1 = a name to locate the constraints file and a PDB file of (dummy) coorinates; 3 = number of cycles (typically 10 to 100, giving 1000 times that number of cycles); 4 = constraints weight (typically 1 to 20).

**Rosetta-TM:** Rosetta also employs an MC-based fragment assembly approach to modelling and it too has a specialiesd TM-protein modelling variant that uses externally predicted helical segment definitions and an estimate of lipid exposure. In addition to these, the pairwise constraints calculated by GREMLIN can be applied as a set of contact distances to the *β*-carbon atoms with parameters calculated by GREMLIN to reflect their degree of reliability. Rosetta was run using the following command line shown in Fig 3, where the .span file is the TM-segment definitions and the the .lips file is the TM-segment lipid exposure estimates calculated by the LIPS method. The increase_cycles parameter, which controls the length of the run, was tested over a range of values from 10 to 100. The constraints file (.cst) was taken from the GREMLIN server and different values of the constraints:cst_weight parameter were tested in the range 1 to 20.

**Fig 4:**
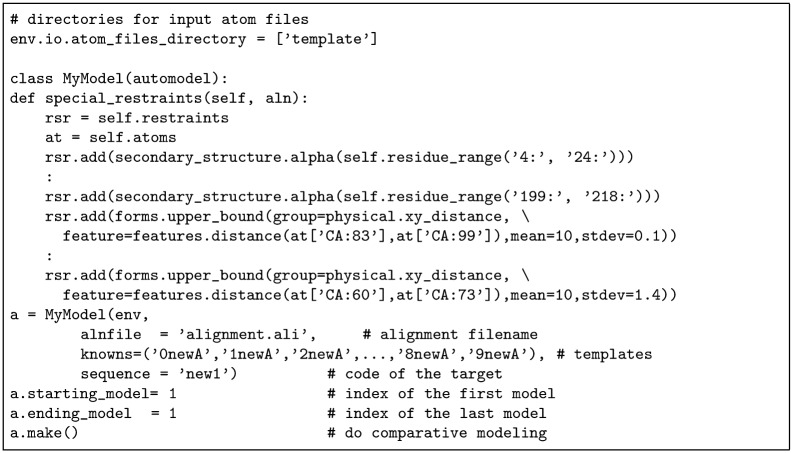
MODELLER runfile as a python script. The template files 0newA’,…,’9newA were model variants generated by the TMpack program, all of which have the same length and sequence. The secondary structure definitions were generated from the TOPCONS output and the constraints were generated from whatever source was being applied (Gremlin, PSICOV or EVfold). Only one model variant is created.

**Modeller/TMpack:** Although Modeller is primarilly used for homology modelling, it has the capacity also to satisfy any set of distance constraints. Compared to the preceeding methods, this is a relatively direct (so fast) calculation but with a conformational search range limited by the degree of variation that is found within the given template structures. To overcome this limitation, we revived an old method (TMpack) that generates a combinatorial selection of ideal folds based on a twisted lattice of helices [48]. Each of these folds was expanded into a range of variations using a coarse-grainned helix packing method [37] to produce a sample of structures that was taken by Modeller as structural ‘homologues’. Modeller takes these variants for each fold as multiple templates and synthesises a combination of their parts that best satisfies the given constraints plus the local helix geometry for each predicted TM-segment. A typical modeler Python command script is shown in Fig 4 which uses 10 template structures selected from the TMpack models that best satisfied the constraints. Only one model is constructed as the variation between multiple modeller models is small compared to the variation between the TMpack models.

### Model evaluation and analysis

Each of the model construction methods incorporate a score based on their own internal ‘energy’ calculations, which makes comparison between models from different methods based on these scores difficult. To avoid this problem, we have assumed that each method produces models with a molecular geometry that is good enough for the rough level of accuracy we require and focused our assessment on the degree to which the model satisfies the given constraints.

**Contact evaluation:** As we are interested only in the path of the chain (the fold), the details of the atomic interactions will be ignored allowing us to focus on the *α*-carbon trace. However, it remains useful to distinguish interactions between one face of a helix and its opposite face, which at the *α*-carbon level is a differnece of only a few Ångstroms. To exaggreate this difference, we extended the *α*-carbon position towards a pseudo-*β*-carbon/centroid location by placing a dummy-atom 2Å beyond the bisector of adjacent *α*-carbon –*α*-carbon virtual bonds. In evaluating a rough model using just a pseudo-centroid position, a strict cutoff distance on a contact definition is not ideal and instead we employed a ‘soft’ cutoff based on a Gaussian function with a maximum at 5Å, which is roughly around the expected minimum separation of the pseudo-centroids in native structures. The strictness with which contacts are evaluated can then be determined by the spread of the Gaussian The inverted distance, *q*, was defined as: *q* = exp((*d* − 5)^2^/*s*^2^), where *d* is the observed pseudo-centroid separation and *s* is the parameter that determines the spread. (*c*.*f*. the standard deviation in the normal distribution).

Given the Gaussian spread parameter, each predicted contact can be scored on the model as a combination of the contact reliability, *p*, (typically a score from 1…0, from strong to nothing) and the Gaussian transformed distance, *q*, (1…0 from ideal…far). A sum of the product of these two values over the set of *N* top predicted pairs produces a score that is highest when the strongest pairs approach the ideal separation. A value of *s* = 5 was used as the spread value which means that separations greater than 10Å will score less than 0.37 and beyond 15, almost zero. Note that separations under 5Å are also slightly penalised with a value of zero also scoring 0.37. The number of residue pairs summed over was taken as *N* = 100 which did not include any interactions with a sequence separation less than 8 (two turns of a helix) to prevent the score being dominated by local interactions. Model rankings were relatively insensitive to the choice of *s* and *N* and variations up and down by a factor of 2 made little difference.

One remaining problem with scoring models across methods was that although the contact reliability values (typically termed a ‘probability’) span the range 0…1, they often have different distributions which will alter the balance of their oveall scores. (Especially the EVfold score which drops off almost exponentially). To reduce this effect, the scores were ranked and converted to a score (*e*) using the Gaussian transformation: *e* = *exp*(−*r*^2^*0.01/*N*), where *r* is the rank of the pair and *N* is the number of residues in the protein. Using this formula, the number of pairs above a fixed value of *e* rises in proportion to the square-root of the protein length.

**Radius of Gyration:** The Radius of Gyration (RoG) is a useful measure of structural compactness often used to evaluate models of globular proteins [49]. For TM-proteins, it is of additional interest to see how well the helices align across the membrane. As the membrane is not represented, we devised a measure that calculates an average axis for the bundle of helices based on their terminal residues (caps), about which an axial RoG can be calculated. Each TM-helix was assigned two capping zones of 4 residues each including, as a minimum, the two terminal residues and the two beyond but extending outwards up to 10 positions in situations where the TOPCONS prediction were variable. Distances between all capping residues were measured and beginning with the most widely separated pair, average axis points were accumulated by adding the remaining pairs in order of separation distance, taking account of orientation. Three RoG values were then calculated about this axis using just the TM-segments, TM+caps (with caps weighted by half) and the whole chain with TM-segments double weight, caps unit weight and the rest half weight. The RoG about this axis is useful mainly to identify models that are not compact. This is a particular problem with methods using a fragment assembly approach (FILM3 and ROSETTA) as often one or two helices cannot be brought into a compact bundle in the allocated time. These then make a very large contribution to the RoG and as a rough guide, any model with an RoG over 15 is likely to have one or more ‘stray’ helices and any model with an RoG over 12 should be checked. These rough guide-lines will vary slightly depending on the size of the protein.

**Root mean square deviation:** Models were compared using the Root Mean Square Deviation (RMSD). However, this is a crude measure based on whole structure superposition and can easily be skewed by a few highly deviant positions (which are not unusual in the rough models constructed below). As the loop regions between the TM-helices are often long and poorly modelled, it is necessary to make allowance for these. Often the TM-align program [50] is used for this type of assessment (the TM here does not designate trans-membrane) but as this is a simplistic approach based on RMSD values over disconnected fragments, we preferred to use the SAP program that calculates a continuous measure of residue similarity based on local environments calculated from their context in the full structure [51]. The degree of similarity in matched environments then provides a weight to apply to that pair in a weighted superposition, from which a weighted RMSD value can be calculated [52]. (*N*.*b*.: there is no alignment problem as all models have a one-to-one correspondance so a version of SAP was used that only calculates the scores of the residue environments for use in the weighted superposition.) In addition to the SAP-weighted RMSD, three RMSD values were also calculated using the weighting schemes described for the RoG values above.

**Fold-space clustering:** The results of the pairwise similarity within a set of structures can be visualised by treating the RMSD values as Euclidean distances and reducing their dimensionaliy to sufficiently few dimensions to be visualised: usually 2 or, better 3, to visualise the space with less distortion. (*N*.*b*.: in theory, pairwise RMSD values are guaranteed to constitute a consistent Euclidean metric but only in N-1 dimensions, where N is the number of structures compared.) Rather than use a simple multi-dimensional scaling (MDS) method ([53]), the more complicated method of multi-dimensional projection was used ([54], see [55] for a simpler exposition). This method reduces the dimensionality of the projection in gradual stages with each step employing triangle-inequality balancing and hyper-dimensional real-space refinement. In the real-space refinement stages, a weight can be applied to pairwise distances. (This cannot be done in direct MDS projection, which can only assign a mass to each point). Weights were assigned to distances as a function of their inverse RMSD, up to a maximum value of 1. The method is robust and has been widely applied to rough models ([56]) and predicted inter-residue distances that constitute highly non-metric data sets ([49]).

## Results

### FlhA

**Fig 5:**
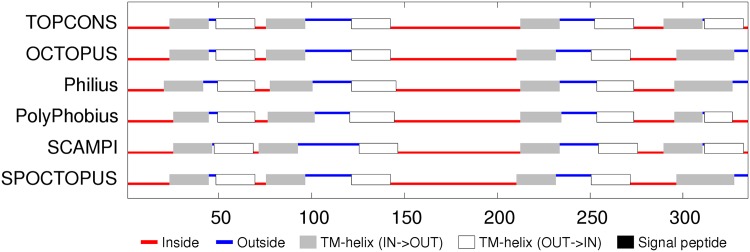
FlhA membrane topology prediction. The summary of the TOPCONS predictions (identified by the name of the method to the right) was taken from the server with its explanatory colouring key. Inside (IN) in the context of the current proteins is the bacterial lumen (cytoplasm) and outside (OUT) is the periplasmic space.

**Sequence analysis:** The 7000 odd sequences of flhA found by JackHmmer were reduced to a representative set of thirty (Fig_S1 in S1 File). The predicted secondary structures were predominantly *α*-helices, eight of which were strongly hydrophobic. The predicted transmembrane (TM) segments were consistent in assigning a cytoplasmic (inside) location for the amino terminus but were equally split on whether the carboxy terminal region should be one or two segments, giving seven or eight segments in total. (Fig 5). Eight segments would agree with the prediction of the two hydrophobic helices seen in the sequence alignment to be spanning this region and would also locate the carboxy-terminus on the inside, where it would be able to connect with the cytoplasmic domain of flhA that forms a visible ring in the electron micrograph images, located on the cytoplasmic side. However, the linker between the transmembrane and cytoplasmic domains is long and could pass through the central pore without exhibiting classic transmembrane characteristics.

**Fig 6:**
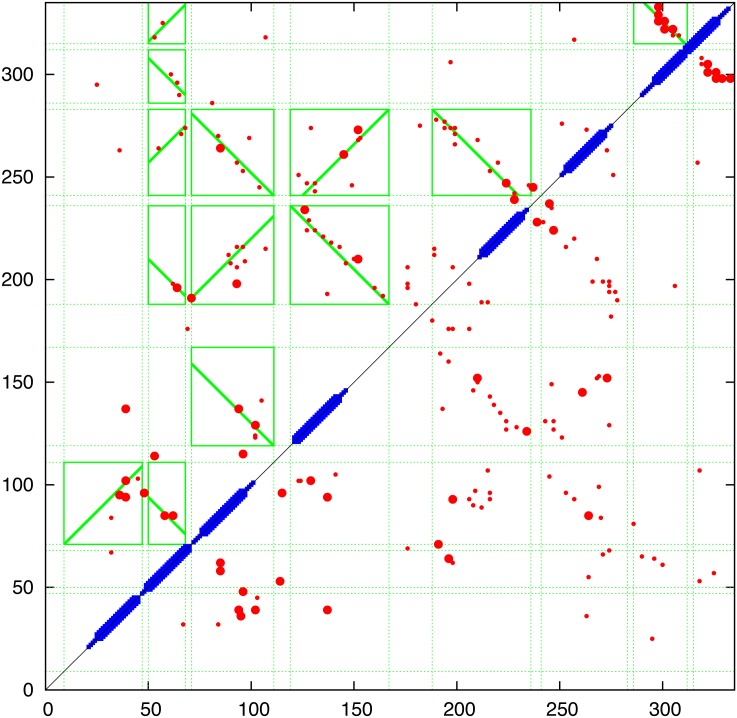
Predicted contacts for FlhA Predicted contacts are plotted as a (symmetric) contact map showing the top scoring 300 contacts (red dots) with the top 50 shown as a slightly larger dot. (Contacts between positions closer that 5 were excluded.) The TOPCONS predition is plotted along the diagonal as a thick bar when all methods agree and a thin bar when two or more agree. The green boxes were calculated by the contact parsing algorithm outlined in the Methods section with a diagonal line indicating the preferred packing orientation. (Note: this always links two opposite edges of the box and do does not always coincide with the best regression fit used in its calculation). The data is a consensus of three prediction methods and individual plots of the component data can be found in Fig_S3 in S1 File.

**Contact prediction:** The contacts predicted using the PSICOV, EVfold and GREMLIN programs are combined in Fig 6 and compared in (Fig_S3 in S1 File). Of particular interest in the evaluation of the predicted contacts was whether there was evidence of the parallel/antiparallel network of interactions what would be expected from aligned helical segments, in contrast to the semi-random ‘tartan’ pattern produced by the selection of generic properties (such as hydrophobicity). Guided by the predicted TM-segments (blue bars on the diagonal in Fig 6), clear regions of parallel/antiparallel packing could be identified in all sets of contacts (upper left) but to a lesser extent in the PSICOV contacts (Fig_S3(b) in S1 File). Interestingly, the interaction ‘stripes’ did not correspond exactly to the predicted TM-segments. This does not necessarily mean the the segment predictions are wrong but may be indicative of helical packing extending beyond the membrane (which is often seen in known structures). A clear example is seen in the contacts predicted between helices 5 and 6 which would correspond with extensions incorporating the predicted non-TM helices seem in the central part of the protein (Fig_S1 in S1 File). In addition, the predicted contacts clearly indicated that the region beyond position 275 forms a helical hairpin, despite being predicted as a single segment by some of the methods included in the TOPCONS server.

To quantify this visual analysis, the contact parsing algorithm described in the Methods section was applied, resulting in the assignment of the stronger interactions indicated by the green boxes in Fig 6. Of the fifteen interactions selected, over half exhibit a clear preference in orientation as indicated by the diagonal line. Applied to the both the EVfold and GREMLIN contacts, the automatic definitions identify a largely alternating pattern of adjacent antiparallel interactions, as would be expected from segments that criss-cross the membrane. The contacts of all three methods were combined giving a consensus prediction and even though the definitions based on PSICOV contacts are slightly less consistent (Fig_S3(b) in S1 File), they contain additional contacts in the loop regions that may provide useful constraints.

**Fig 7:**
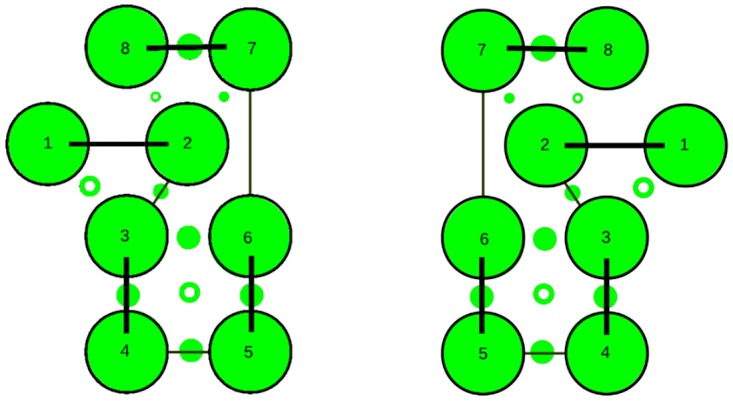
Summary of helix packing interactions for FlhA. The eight predicted helical TM-segments for FlhA (numbered 1…8) are arranged in a configuration that accounts for most of the interactions seen in the contact plots. These are represented by small circle between the helices with a solid circle indicating an antiparallel, and an open circle representing a parallel interaction. The two mirror-image depictions emphasise that distance constraints do no discriminate chirality at this level of representation.

The resulting consensus was completely consistent with the packing of helices that pass alternatively up and down across the plane of the membrane with no evidence of re-entrant helices (or parallel interactions made by extended regions passing through a pore). The thirteen interactions boxed on the consensus assignment (Fig 6) imply that all helices are contacting in a single bundle with a core consisting of helices 3 to 6. Helices 1 and 2, wrap around this core and do not pack with each other while helices 7 and 8 pack strongly as a hairpin (containing the strongest predicted residue pairings in all methods) but only interact weakly with the main body of the protein through helix 2. These contacts are summarised in the possible packing arrangement shown in Fig 7. Other possible configurations will be considered below but it should be remembered that, at this level of resolution, it is almost impossible to distinguish mirror-image configurations. This applies also to the relative orientation of sub-domains that are linked through a single pivot. So if the weak interactions to helix-1 are ignored, helices 7 and 8 can swap places. (*N*.*b*.: reference to a mirror image configuration does not imply that the alpha helices have flipped their hand and in the molecular models described below, these all retain their native chirality. However, in rough models, the small contribution from *α*-helix chirality may not be sufficient to discriminate between higher level difference in packing chirality.)

**Structure prediction:** The interactions identified in the previous section are sufficiently specific to allow the construction of a three-dimensional model. As outlined in the methods section, models were constructed using a variety of methods as *α*-carbon backbone traces and all models were evaluated and ranked using each of the three sets of predicted contacts under the common scoring scheme described in the Methods section.

**TMpack:** The top 50 models generated by enumeration of possible helix packing configurations were refined at a coarse-grainned level using different sized sets of constraints ranging from 90 to 180 in steps of 30. The resulting models were then recombined using the program Modeller (as described in the Methods Section) giving a set of 200 models. Each model was scored using the soft contact evaluation score and plotted against the radius of gyration (RoG) about the axis of the helical bundle (as defined in the Methods Section) using each of the three sources of predicted contacts, along with a combination of all three. (Fig_S4(a-d) in S1 File, red dots).

**FILM3:** One thousand models were generated using the FILM3 program with run lengths of 20, 30 and 50 million cycles in the ratios 1:3:1, respectively. Compared to the helix-packing approach (which generates only compact models), the stochastic assembly approach of FILM3 generated many models that had helices detached from the core bundle. This is reflected in the greater range of RoG values. However, the best scoring models were comparable to those obtained with helix-packing. (Fig_S4(a-d) in S1 File, green dots).

**Rosetta:** Initial trials of the Rosetta method, which uses a stochastic assembly approach similar to FILM3, resulted in even less well packed models with lower scores. To see if this was simply the result of poor sampling, 20,000 models were generated. Only the tip of this ‘iceberg’ is plotted in (Fig_S4(a-d) in S1 File, magenta dots).

**EVfold:** The EVfold server generates models using various sized subsets of constraints and all of these were re-score so as to make them directly comparable with the previous methods. Although only 50 models were available, they span a range intermediate between the Rosetta and the helix-packing models. (Fig_S4(a-d) in S1 File, blue dots).

### Model selection

**Fig 8:**
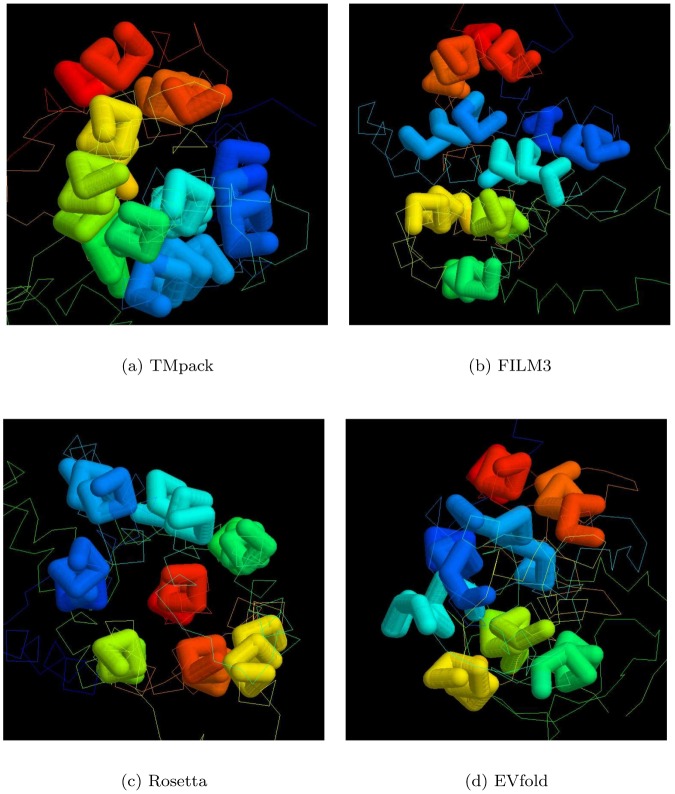
Core helix packing for top models. The *α*-carbon traces of the top scoring models generated by each method (*a*…*d*) were viewed down the axis of their helix bundle with just the central portions of the helices rendered as a thick tube (in RASMOL), with the remaining structure left as a thin line. The helices are coloured from blue (amino terminal) through spectral hues to red (carboxy terminal).

**Top-slice consensus:** To retain a representative sample from each modelling method with each constraint source, a diagonal slice was made across the plots in Fig_S4(a-d) in S1 File with a gradient *y* = 10*x* + *c* with the constant *c* chosen in each case to select up to 20 models that lie in the upper-left of the plot: being those with the highest score and/or the lowest RoG. The selected models were then further culled by selecting only those that occur in at least three of the slices. This reduced the models to 54 comprising 9,13,15,17 for each of the methods: FILM3,TMpack,Rosetta,EVfold, respectively. The top-scoring model for each of the methods using the combined constraints was selected as a representative of each set and their helix packing examined. Over the core residues, there was little in common between these models with no pairwise RMS deviations under 10Å, even using the tolerant SAP-weighted score. Visulaisation of the central segments of the core helices showed various packing arrangements (Fig 8).

Of the four models shown in Fig 8, the FILM3 configuration of helices has the closest similarity to the idealised packing arrangements shown in Fig 7(*right*)), with just the positions of the core helices 4 and 5 swapped. The TMpack solution keeps the four core helices as a simple bundle (as in Fig 7(*right*)) but helix-2 is displaced from the interface between the core and the 7-8 hairpin. The EVfold model has the same core arrangement as FILM3 and retains helix-2 as an interface to the 7-8 hairpin but in a distinctly different arrangement. The Rosetta model has the 7-8 hairpin deeply buried—which is a configuration that seems difficult to reconcile with any rearrangement of the pairwise interactions summarised in Fig 7.

**Fig 9:**
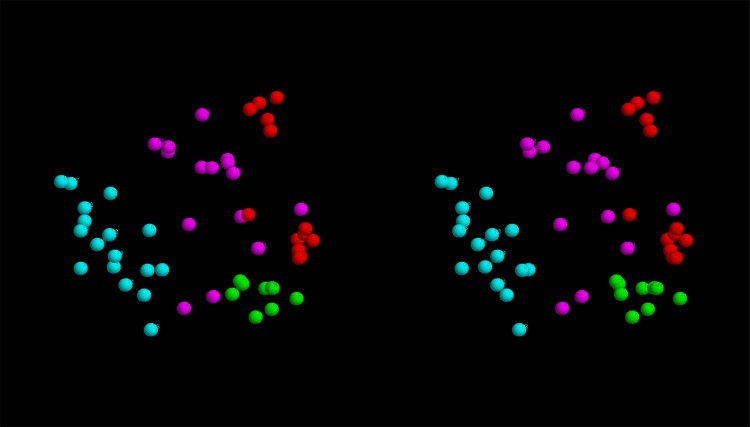
Fold-space representation of top-scoring FlhA models. The best models from each method were projected into a 3D fold-space as described in the methods with models from: TMpack in red, FILM3 in green, Rosetta in magenta and EVfold in cyan.

**Structural clustering:** To see if there was any consensus among the top scoring models, these were all compared pairwise with their similarity measured using the SAP-weighted RMSD. The resulting set of RMSD values were treated as Euclidean distances and embedded into a 3-dimensional space as described in the Methods section. The resulting projection shows that models tend to cluster by the method that generated them, either as broad scatterings (Rosetta and EVfold) or as a single relatively compact cluster (FILM3) or as two separate clusters (TMpack). (Fig 9). The latter bifurcation is a consequence of the combinatorial enumeration folds that results in roughly equal numbers of folds and their mirror-images. (As depicted for the idealised example shown in Fig 7).

Despite the good preservation of distances using the gradual projection algorithm, some distortion remains, especially involving the weaker similarities between models derived from different methods. However, by considering the ranked list of similarities, all-bar-three of the 25 closest pairs between models from different sources are between the FILM3 models and the larger TMpack cluster (located towards the lower right of the fold-space image in Fig 9). This joint cluster, which also incorporates a few Rosetta models can be summarised by the simple network:

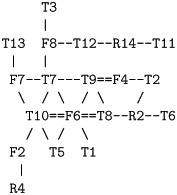

Where T, F and R represent TMpack, FILM3 and Rosetta models, respectively, with “==” linking the most similar pairs.

The heart of this network is formed by the TMpack model ranked 10 (T10), FILM3 model 6 (F6) and TMpack model 8 (T8). The superposition of the F6 model with all its adjacent TMpack models showed little discrimination using an (unweighted) RMSD measure but visual inspection revealed that only the T10 model had the same packing arrangement as its FILM3 neighbours, with just a change in relative tilt of the 7-8 terminal hairpin. All the others had helices in swapped positions, which is a change that is also difficult to detect even in smaller globular proteins using an RMSD metric [57]. The three core FILM3 models all had the same fold which was a good approximation to the right-handed enantiomer depicted in Fig 7(right). The cumulative RMSD values from their superpositions with each other (green) and their neighbours in the network, including T10 (blue), are plotted in (Fig_S5(a) in S1 File).

**Fig 10:**
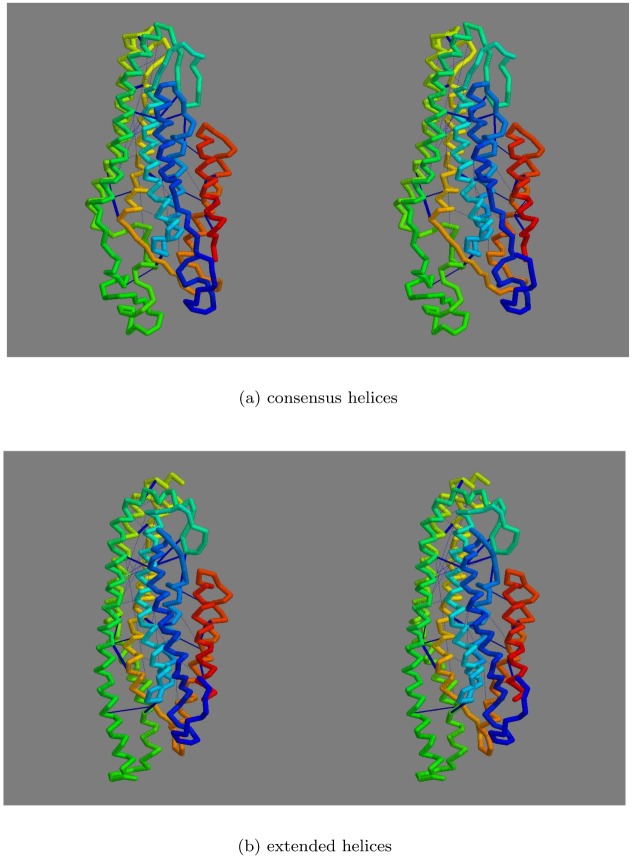
Final FlhA model. The final consensus model for FlhA is shown in frame *a* as a stereo pair, coloured from blue (amino) to red (carboxy terminus). The orientation is such that the cytoplasmic globular domain would lie (well) below. Part *b* shows the model constructed but with the helices extended into the regions suggested by the contact-parsing program. The strongest predicted contact pairs are linked by dark-blue bars.

**Consensus model:** The convergence of the FILM3 and TMpack methods on a common solution that corresponds closely to what might be expected from the simple analysis of helix packing (Fig 7) opens the possibility to construct a consensus model. To do this, the same Modeller protocol that was used by TMpack was employed (Fig 4) but using a database of “homology” models consisting of the three FILM3 models (F4, F6, F8) that form the core of the FILM3/TMpack cluster (Fig 9), along with three variants of the TMpack model (T10), generated with 120, 150 and 180 constraints. When examining the origin of the T10 model, it was found to have converged towards the FILM3 models from a starting configuration in which helices 7 and 8 were farther apart and helix 1 and 2 were displaced to the side (adjacent to helices 3 and 4 in the configuration of Fig 7). Investigation of why a better starting configuration had not been used, revealed that the configuration closest to that in Fig 7 had fallen below the score cutoff used to select the top 50 folds. For completeness, the packing represented in Fig 7 were manually entered and three variants of the corresponding model were selected and added to the mix for Modeller to use. The resulting consensus structure produced by Modeller from this collection was a good representation of all the contributions as can be seen from the cumulative RMSD plot (Fig_S5(b) in S1 File). Two variants of the final consensus structure were generated using Modeller, one with the ‘default’ transmembrane helical segments and an alternative with the long loop regions modelled as helical extensions of the TM-segments (Fig 10).

### FlhB

**Fig 11:**
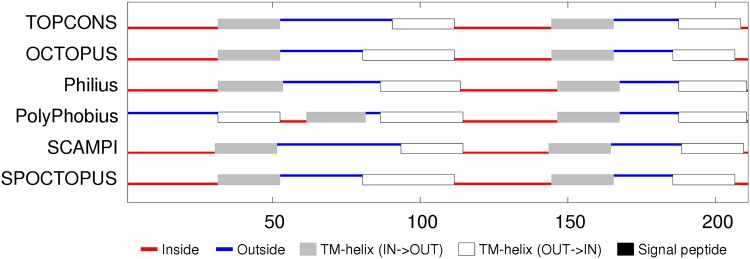
FlhB membrane topology prediction. Bar one, the methods employed in TOPCONS server are consistent in their predicted topologies. (For details, see legend to Fig 5).

**Sequence analysis:** The reduction of the flhB sequences to a representative set indicated four main helices, each of which were, to varying degrees, predicted as TM segments (Fig_S7 in S1 File). Smaller helices were predicted in the central region and at the amino-terminus. Being deleted in some of the sequences, the latter may be a signal peptide but is very hydrophilic and is not identified as such by the TM prediction methods, which predict predominantly four segments, oriented with the amino terminus on the inside (cytoplasmic). (Fig 11). The two halves of the flhB sequence are sufficiently similar that they were aligned to each other to check for any matching motifs. (Data not shown). Although there was little except the generic sequence correspondence expected from two similar sets of secondary structures, it would not be difficult to imagine that the protein had its origin in an ancient gene duplication event.

**Contact prediction:** The contact predictions by all three methods were remarkably consistent, and as such, only the GREMLIN result is shown as a representative, along with the consensus of all three methods. These contacts predict two long and strong helical hairpins formed by helices 1+2 and 3+4 but with only a single clear additional packing between helices 1 and 4. This would imply a less compact packing arrangement, which is consistent with the greater degree of variation seen in the multiple sequence alignment.

**Fig 12:**
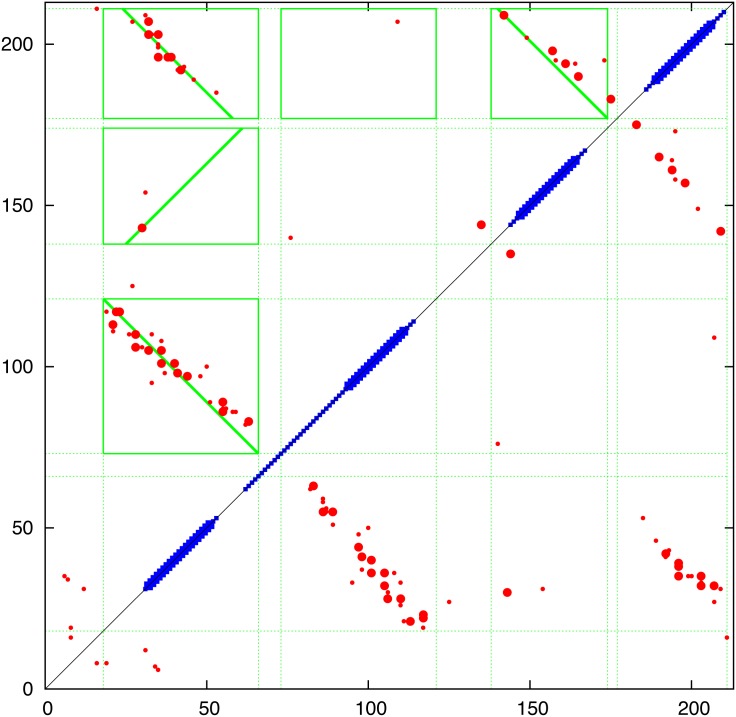
Predicted contacts for FlhB are plotted as in Fig 6. (See Fig_S9 in S1 File for individual plots).

**Structure prediction:** Given the exceptionally clear contact prediction for the flhB sequence over all methods, there is effectively only one possible packing arrangement, which is a pretzel-like arrangement of the helices bringing helices 1 and 4 together in an open structure with their termini entwined (as indicated by the contacts in the lower-left of the contact matrix (Fig 12). However, there are a few scattered contacts involving the start of helices 1+3 and the (C-terminal) ends of 2+4. This suggests a more compact packing at one end of the molecule rather like the catcher’s glove in the game of base-ball. This raises the problem of whether it is a right or left handed glove. (*N*.*b*.: as with any set of distance constraints, it should also be remembered that a multimeric domain-swapped configuration can account equally well for the data. However, following William of Occam, we will restrict our immediate attention to monomeric solutions.) Following the approach used with the flhA protein, to elicit a preference between these enantiomers, the models generated by FILM3, TMpack and EVfold were clustered. (Given the amount of computing time used by the Rosetta method to produce ambiguous results with flhA, this method was avoided.) As above, those with the best combinations of score and compactness under each of the prediction methods (and their combination) for each of the modelling methods, were top-sliced into a pool of best models. However, given the narrower range of scores in this smaller protein and its consistent set of contacts, a single cutoff line was used for each set of contacts (Fig_S11(b) in S1 File).

**Fig 13:**
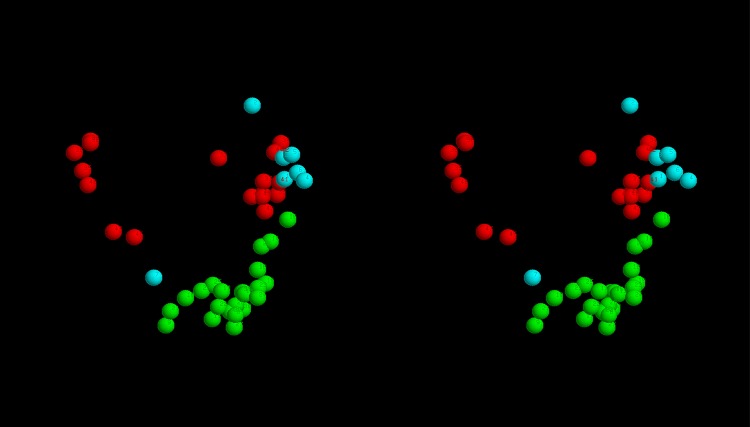
Fold-space representation of top-scoring FlhB models. The best models from each method were projected into fold-space (see Methods) with models: TMpack = red, FILM3 = greenand EVfold = cyan.

The models that passed the “cut” were then compared pairwise and plotted in fold-space. (Fig 13). The FILM3 method favoured open (pretzel) structures whereas, with its origin on a compact lattice, the TMpack method preferred 4-helix bundles and the EVfold method lay somewhat between. By systematically enumerating packing arrangements, the TMpack method generated bundles of each chirality and when plotted in fold-space, these (literally) depict the dilemma of enantiomer choice as a pair of ‘horns’. As viewed in Fig 13, the clockwise packing lies to the right and anticlockwise to the left (as viewed down the bundle with the first helix approaching). These are linked by a scattering of other models but there is a clear asymmetry, especially in the EVfold models, suggesting a preference towards the anti-clockwise configuration.

**Fig 14:**
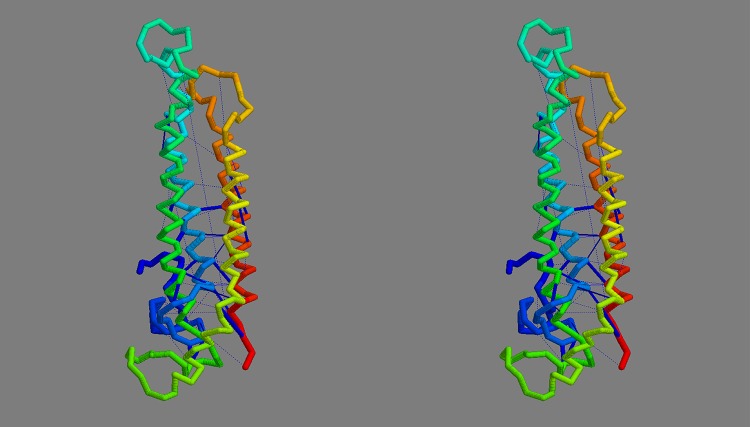
Final FlhB model. The final consensus model for FlhB is shown as a stereo pair, coloured from blue (amino) to red (carboxy terminus). The interior of the cell would lie below the model. The cumulative RMSD plots of the consensus model against the set of models from which it was constructed by Modeller can be seen in Fig_S11(b) in S1 File.

Six structures were selected from the region in fold-space where the three methods converge. These were compared pairwise and were found to be similar enough to be combined using Modeller. The resulting consensus structure (Fig 14) is a good fit to all its constituent component structures used by Modeller (Fig_S11(b) in S1 File) and also to the constraints. However, the lack of constraints between helices 2 and 3 (green and orange in Fig 14) means the structure may be more open towards the top (as viewed in the figure).

### FliP

**Fig 15:**
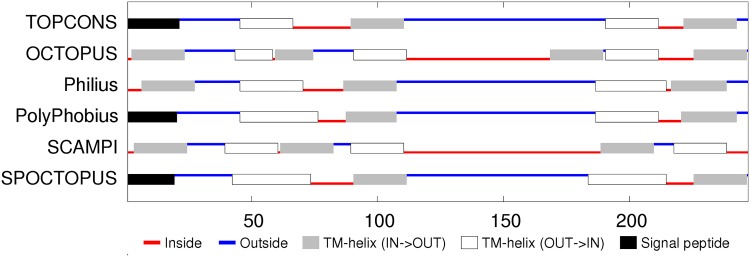
FliP membrane topology prediction. (For details, see legend to Fig 5).

**Sequence analysis:** Like the flhB sequences, two pairs of TM helices are predicted in the amino and carboxy regions but with a more extensive non-TM helical region lying between them with the possibility that the carboxy terminus also forms a separate shorter helix (Fig_S12 in S1 File). The TM predictions show some variation but overall are consistent with the four TM segments with the shorter C-terminal helix being part of the final segment. The variation in the methods is partly caused by the ambiguous interpretation of the N-terminal region either as a TM segment or a signal peptide. (Fig 15).

**Fig 16:**
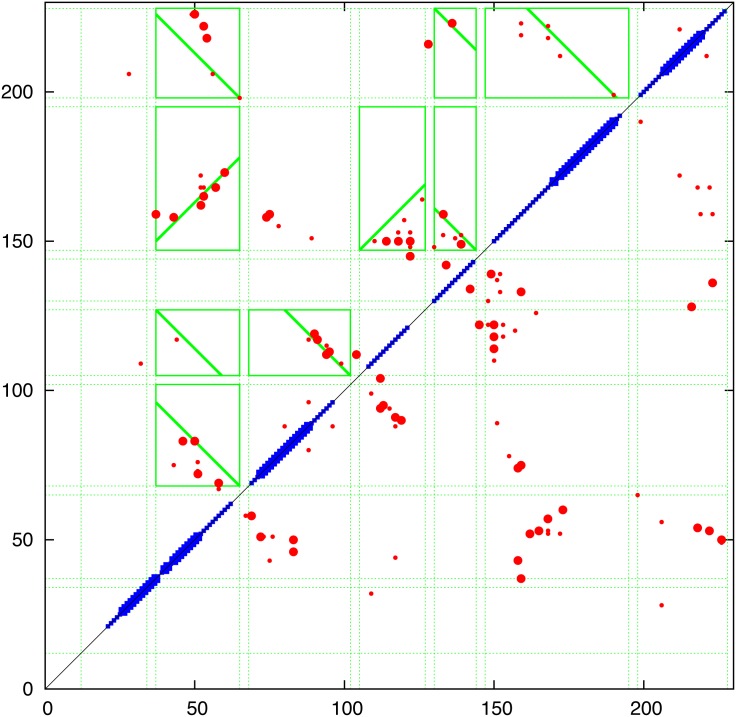
Predicted contacts for fliP (7 helices) plotted as in Fig 6. (See Fig_S15 in S1 File for individual plots).

**Contact prediction:** Interpreting the contact predictions in terms of the four predicted TM-segments gives an incomplete picture, with inconsistent adjacent packing orientations indicated and a number of strong predicted contacts unaccounted for (Fig_S14 in S1 File). However, adding two additional segments to correspond to the helices predicted in the non-TM middle segment of the sequence and splitting the first TM-segment generated a more coherent assignment (Fig 16). The observed pattern of contacts suggest two TM helical hairpins 1+2 and 3+4 with the non-TM helices forming interactions between these pairs at a level above the plane of the membrane. Within the membrane, the stronger interactions between the hairpins involve mainly helix-1 but in the half that is closer to the hairpin, leaving the first half of the helix with almost no interactions. This mis-match of levels is investigated below using 3D modelling.

**Fig 17:**
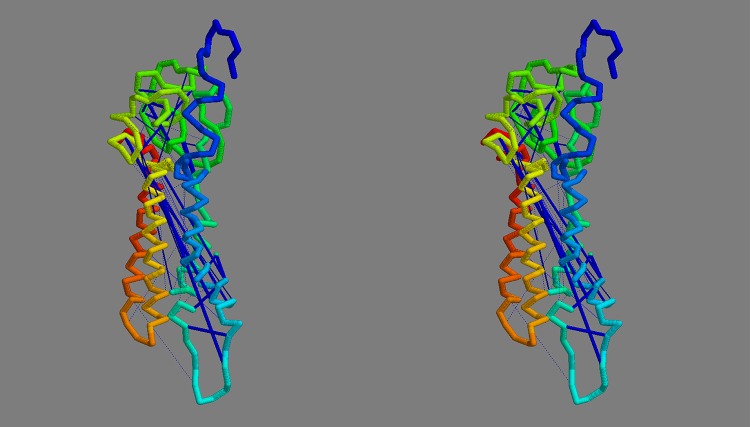
Four-helix bundle FliP model. Although the helix packing results in a compact structure, many predicted contacts are too long (dark blue rods). These could be improved by flipping the orientation of the terminal pair of helices (yellow–red) but this would place the carboxy terminus on the side of the membrane where predictions and experiments indicate it should not be found.

**Structure prediction:** The packing analysis of the previous section suggests that the structure of fliP might resemble another 4-helix TM-bundle with some additional helical segments packing in the link between TM-segments 2 and 3. However, a simple model constructed along these lines indicated that extensive predicted contacts run along the bundle axis. (Fig 17). The predicted contacts that lie at opposite poles of the model can be easily brought into a reasonable range by flipping the 1+2 segments relative to the 3+4 segments. However, this arrangement is less consistent with the membrane topology predictions that indicate a periplasmic location for both the amino and carboxy termini (See ref. [58] and Fig 15).

An alternative solution that maintains the in/out polarity of the helices is to introduce a large displacement between the 1+2 and 3+4 TM segments. As this is too large a shift for TMpack to explore from starting models like that in Fig 17, this was introduced manually by redefining the helical segments that should lie in the plane of the membrane. Both FILM3 do not have the constraints of a starting lattice so are free to explore the possibility of such a shift.

To allow scope for conformational exploration, 100 models were generated with FILM3, running for 20 million cycles, using each of the sets of predicted contacts from gremlin, psicov and EVfold (producing 600 models). Similarly, TMpack was run with each of the sets of contacts and their combination, generating 200 structures. The EVfold server returned the default 50 structures. These were all scored by each method and filtered as with the previous proteins to retain models that were compact and/or high scoring (Fig_S17 in S1 File).

**Fig 18:**
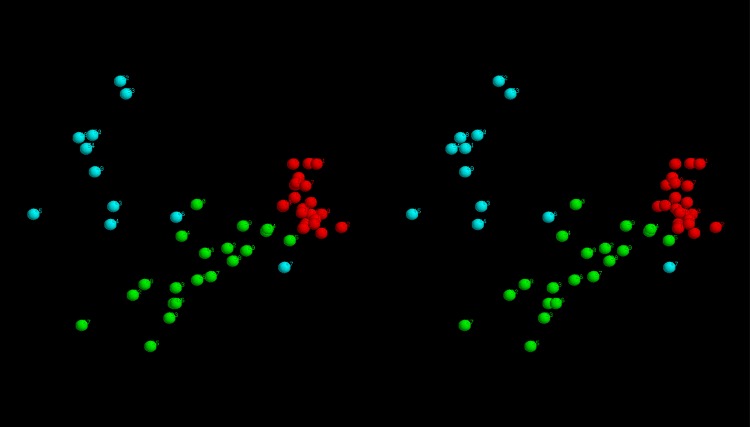
Fold-space of top-scoring FliP models. The best models from each method were projected into fold-space (see Methods) with: TMpack red, FILM3 green and EVfold cyan.

**Fig 19:**
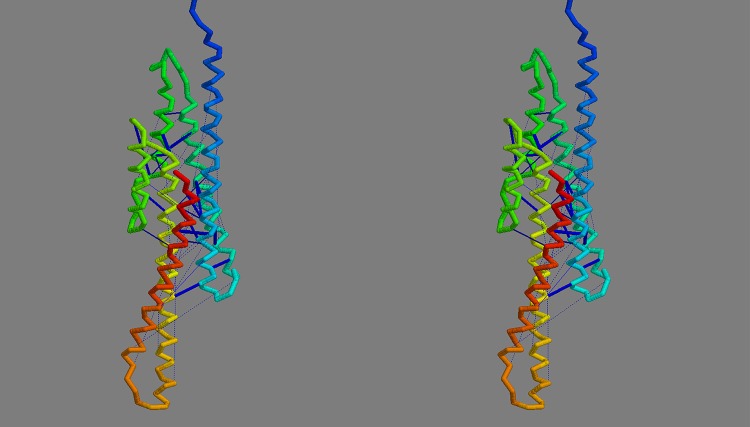
Final FliP model. The final consensus model for FliP is shown as a stereo pair, coloured from blue (amino) to red (carboxy terminus). The interior of the cell would lie below the model. Compared to Fig 17, the contacts (dark blue bars) are now all much shorter. The cumulative RMSD plots of the consensus model against the set of models from which it was constructed can be seen in Fig_S18(b) in S1 File.

To retain a reasonable sample of EVfold models, the cutoffs were reduced and the excess FILM3 and TMpact models culled by discarding those that recurred least often. Those remaining were then compared pairwise and plotted in fold-space. (Fig 18). In this it can be seen that the TMpack solutions (red) are all similar and are adjacent with the more extended FILM3 cluster (green). Four models from each method were selected at this interface, along with the EVfold outlier lying towards the right side of the plot. From a visual assessment of their superposition, these models all had a similar core topology and were passed to Modeller to construct a consensus model. (Fig 19).

The consistency of the final consensus structure was assessed by comparing it with each of the models used by Modeller. This revealed one of the FILM3 models to have a high deviation and the consensus was regenerated without it, producing the more consistent result shown in Fig_S18(b) in S1 File.

### FliQ

**Fig 20:**
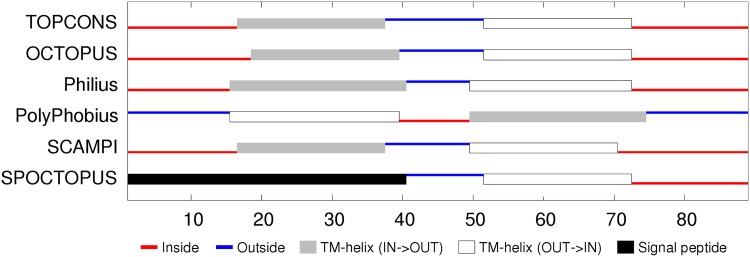
FliQ membrane topology prediction showing a consensus for cytoplasmic termini. (For details, see legend to Fig 5).

**Sequence analysis:** In contrast to the preceding proteins, FliQ is rather simple: having two predicted helical segments, and although these tend to merge in the psipred predictions, there is a clear region of more negatively charged residues between them (Fig_S19 in S1 File). This prediction is supported by the TM predictions which indicate two TM segments with a topology placing the termini inside. (Fig 20).

**Fig 21:**
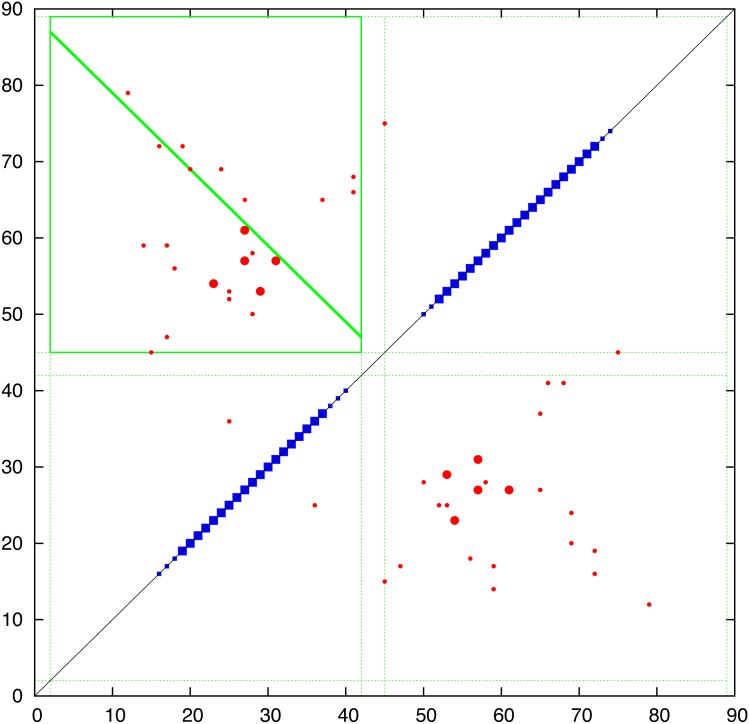
Predicted contacts for FliQ plotted as Fig 6. Although simple, all methods show a consistent birfucation of the interaction (Fig_S21 in S1 File).

**Contact prediction:** The contact predictions confirm the helical hairpin packing suggested by the sequence analysis and are consistent across the three methods (Fig 21). When examined individually, the plots show an unusual and consistent bifurcation of the contact stripe (Fig_S21(a-d) in S1 File). The different slopes of the two contact stripes could be explained by two distinct packing angles between the helices. This could be explained either by the co-existence of two conformations, by a dynamic transition between two conformations or by a multimeric interaction that has a different packing from the intra-molecular packing.

**Structure prediction:** Given the clear and trivial nature of the fliQ contacts, a molecular model was not constructed.

### FliR

**Fig 22:**
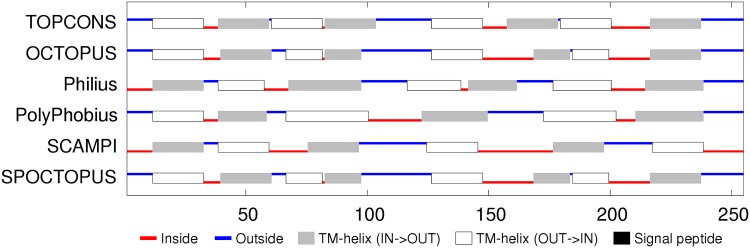
FliP membrane topology prediction. (For details, see legend to Fig 5).

**Sequence analysis:** FliR returns again to a more complex situation with seven predicted helices, six of which are potentially TM segments (Fig_S22 in S1 File). This ambiguity is compounded by the TM topology predictions which range from six to eight segments with a variety of in/out topologies. (Fig 22). The level of sequence conservation is not high (beyond what is expected for alternating hydrophobic and polar segments), however there are a few almost absolutely conserved positions, including an arginine in the middle of the first TM-segment, a glycine in the third segment and a proline in the fifth. These positions will be re-examined below in the light of a predicted structure. The only other feature of note is that the sequences contain an unexpectedly high proportion of methionine residues, with over 10% in the *Campylobacter* sequence modelled below, twice as many as alanine. However, no positions exhibit any marked methionine conservation.

**Contact prediction:** The interpretation of the predicted contacts using the consensus MEMSAT predictions (Fig_S22 in S1 File). is, in contrast to the topology predictions, remarkably consistent. Although the PSICOV contacts appear to be more ‘noisy’, the consensus of all three methods predicts a consistent packing with strong packing between helices 1-2-3 (but not 2-3) and hairpins formed between 3-4 and 5-6 but little between 4 and 5. There is some suggestion of contact between 6 and 3 but only in the 3-4 + 5-6 connecting loop region. These interactions, which lack contacts involving the amino and carboxy helices, suggest a more extended structure, with the helices packed in staggered zig-zag like arrangement. Like the less compact FlhB, this would be consistent with the higher degree of amino acid variation seen in the multiple sequence alignment (Fig_S22 in S1 File).

**Fig 23:**
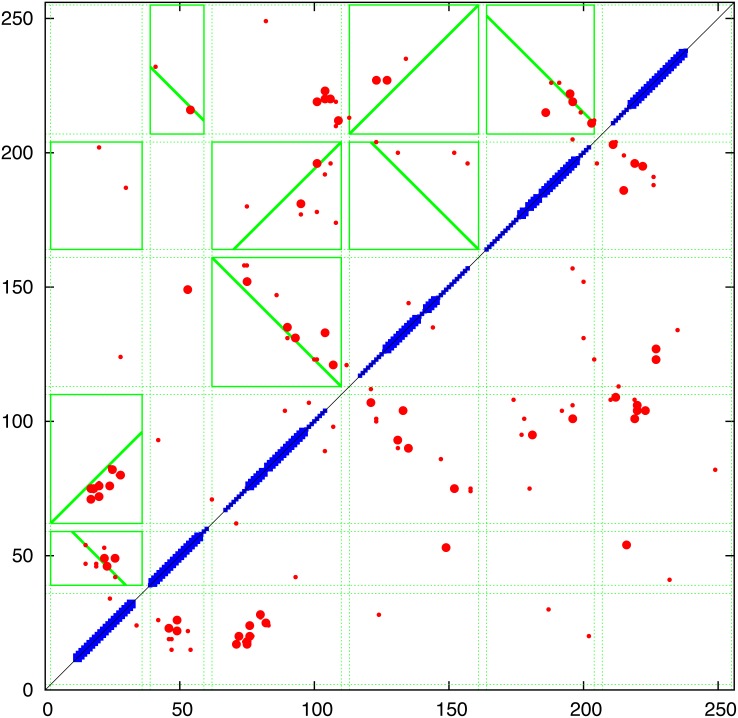
Predicted contacts for fliR plotted as in Fig 6. (See Fig_S24(a-d) in S1 File for individual plots).

**Structure prediction:** As above, the structures were predicted using the FILM3 program, constructing 100 models with the ‘default’ 20 million cycles for each of the three predicted sets of constraints (giving 300 models). The TMpack/Modeller combination was run initially using the default TOPCONS definitions with the 60 top constraints from each of the three constraint sets. However, visual examination of the models suggested that the models would be better packed if the helical definitions were extended into the boxed segments suggested by the contact parsing algorithm (Fig 23). With these definitions, a further set of models was constructed both using 60 and 120 constraints, giving in total, 300 models. The EVfold server returned the default 50 structures.

All these models were scored by each method (and their combination) and filtered as with the previous proteins to retain models that were compact and/or high scoring (Fig_S25 in S1 File). For each scoring scheme, this revealed a distinct segregation between high-scoring but less compact FILM3 derived models with more compact but sometimes lower scoring TMpack models. The EVfold models were neither high-scoring nor compact. This may be a consequence of under-sampling, but rather that try to maintain an even balance of models between methods, they were in this case discarded. (*N*.*b*.: the term “compact” refers to the radius-of-gyration about the axis of the bundle of helices. As will be discussed below, some of these structures are indeed compact but have an ill-defined bundle axis.)

**Fig 24:**
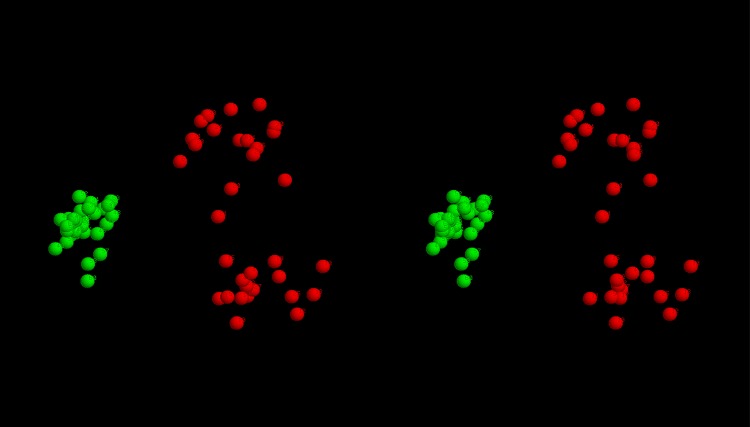
Fold-space of top-scoring FliR models. The best models from each method were projected into fold-space (see Methods) with: TMpack red, FILM3 green.

The FILM3 and TMpact models were culled by discarding those that recurred least often across all the scoring schemes to give an even balance of close to 30 models from each method. Theses were compared pairwise and plotted in fold-space. (Fig 24). In this it can be seen that the FILM3 solutions (green) are all very similar whereas the TMpack models (red) form a half-circle reflecting the clockwise/anti-clockwise packing of the four carboxy-terminal helices. Unlike previous fold-space projections, the FILM3 models remain equidistant from this ring.

The divergence of the FILM3 and TMpack models derives from the distribution of predicted contacts between the two ends of the packed helices, in which helices 1, 2 and 3 make extensive contacts at one end whereas the contacts between helices 4, 5 and 6 cluster at the opposite end. With no constraint to cross a membrane these two domains were free to bend/twist towards each other whereas in the TMpack models they are confined to opposite ends. In general the FILM3 Z-filter was not applied as displacements, such as those seen in FliP, would have been excluded on the basis of the TOPCONS or MEMSAT predictions which are more simplistic methods that does not take account of 3D packing.

As this distinction is not topological, there exist intermediate structures with some degree of similarity. As with FlhA, the most similar pairings between methods can be arranged in a simple planar network:

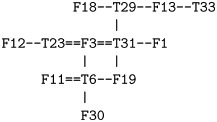

(where Fn is a FILM3 model and Tm a TMpack model and ‘==’ links the most similar pairs).

**Fig 25:**
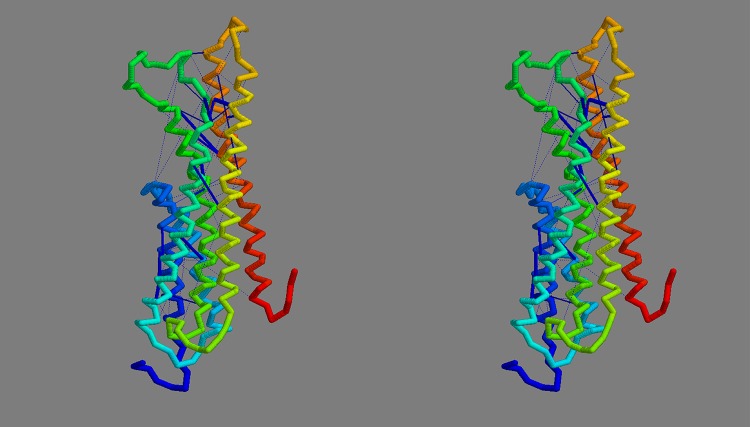
Final FliR model. The final consensus model is shown as a stereo pair, coloured from blue (amino) to red (carboxy terminus). The interior of the cell would lie below the model. The cumulative RMSD plots of the consensus model against the set of models from which it was constructed are in Fig_S26(b) in S1 File.

From a visual assessment of their superposition, the core of this network (T23,F3,T31,F11,T6,F19) had a similar core topology and were passed to Modeller to construct a consensus model. (Fig 25) and the comparison of this to its source components revealed a close and evenly spread similarity (Fig_S26(b) in S1 File). The consensus model retained the more linear arrangement of the TMpack models but it should be borne in mind that in a complex protein/lipid environment that incorporates a pore, the conventional expectation that extended trans-membrane helices should remain clear of the membrane level, may be broken.

The junction between these extensions and the main TM-level also includes the highly conserved proline (P:178) supporting a more kinked conformation. A second conserved proline just at the end of helix-1 and probably provides a “helix-breaker” function. The other two highly conserved positions (R:21 and G:79) pack exactly adjacent, with a separation of 5Å between their *α*-carbons, at the mid-level of the main TM bundle.

## Discussion

### Structural Analysis

**Distribution of hydrophobic residues:** Compared to conventional structure prediction methods, an unusual aspect of our models is that the physico-chemical nature of the amino acid sequence played no part in their construction (beyond the identification of TM segments) as all the covariance analysis methods are ‘blind’ to the chemical nature of the letters that constitute the multiple sequence alignment. This means that the disposition of hydrophobic and polar residues in the models is unbiased by their properties and can therefore be used as an independent check on which regions of the surface may be exposed or buried or interfacing with membrane lipids.

Both FlhB and, to a lesser extent, FliR have a belt of hydrophobic residues in their central zone that is characteristic of simple transmembrane proteins. In FlhB, a pair of polar residues (GLN 30 and LYS 150) slightly encroach on this region but are oriented towards the protein interior and are close enough to form hydrogen bonds. FliR has more polar residues in the TM-belt region and of those in the protein interior, most are histidines. However, as these positions are not conserved in the multiple sequence alignment, it is unlikely that they are of any functional significance.

FlhA’s core is composed of helices 2, 3 and 6 and contains a few polar and fewer charged residues, however, the adjacent portions of helices 4 and 5 and their connecting sequence contain many charged and some polar residues. This region is well conserved and has been the subject of genetic studies [59, 60]. In particular, ASP 208 is juxta-membrane on helix 5 and has been hypothesised to bind a proton as part of the proton influx pathway. Further from the membrane, LYS 203 may interact with the first cytoplasmic loop of FliR as suggested by suppressor mutations which partially restore motility [59]. VAL 151, also in the loop, may have a role in substrate selection [60].

If it is assumed that the TM-belt in FlhA corresponds with the highly hydrophobic pair of C-terminal helices, then a number of charged positions would be predicted to lie in the membrane zone. These fall towards one side of the helical bundle in helices 4 and 5 and, to a lesser extent, 6. While it is possible that this region may face a solvent accessible pore, the mutation studies mentioned above suggest that this collection of charged residues may constitute the proton channel that drives secretion. Although FlhA is the most likely candidate for the proton translocation function, being the most abundant protein in the export gate [61] and essential for secretion [59, 62], a complete transmembrane proton conduction pathway is not obvious in our model.

FliP has the least conventional distribution of polar residues on its surface with one side of the molecule containing a high density of polar and charged residues. These are associated mainly with and around the shorter helices located in the mid-region of the sequence (coloured green in Fig 19) and predicted in our model as re-entrant helices (i.e., not transversing the membrane). As this conformation is well tethered by predicted constraints, it seems unlikely that they can be relocated away from the TM-belt, thus making this the most likely part of the molecule to be facing towards a pore.

The C-terminal helical hairpin which extends beyond the postulated membrane belt is remarkably hydrophobic for its exposed location in our model. This suggests that it may lie in a buried environment possibly created by multiple copies of FliP around the pore. This would create a ring of the highly conserved methionines that lie in the loop connecting the two helices, perhaps contributing to a gate-keeper function (Marc Erhardt and Thibaud Renault, personal communication).

**Implications for complex assembly:** The manner in which our five component proteins assemble into a pore complex remains unclear and is hampered by a lack of clear experimental evidence, with more reliable estimates of stoichoimetry only available recently [61]. A ring of nine FlhA molecules has strong support including structural confirmation of the nonameric packing of the FlhA cytoplasmic carboxy terminal domain [63], while the recent results suggests that there may be a ring of five or even six FliP molecules, probably fitting inside the FlhA nonamer, with the other components being present in low copy numbers or as singletons [61]. Such an arrangement, placing FliP at the core inside a ring of FlhA, would be consistent with our predicted models but the location of the other components relative to this will require more experimental constraints.

### Conclusions

We have shown that by using a combination of computational methods, consistent three-dimensional molecular models can be proposed for the core proteins of the type-III secretion system (T3SS). We employed a variety of approaches to reconcile disparate, and sometimes inconsistent, data sources into a coherent picture that for most of the proteins indicated a unique solution to the constraints.

The range of difficulty spanned from the trivial (FliQ) to the difficult (FlhA and FliP). The uncertainties encountered with FlhA were largely the result of the greater number of helix packing possibilities allowed in a large protein, however, for FliP, there remains an uncertainty in how to reconcile the large displacement predicted between its two main helical hairpins and their ability to sit together ‘happily’ across the bacterial inner membrane.

Our predictions were all made on the basis that these proteins exist in the context of a conventional lipid bilayer. To a first approximation, this is supported by the hydrophobic nature of the predicted transmembrane segments, however, it is known that these proteins exist and function as a macromolecular machine that has a high protein density and includes a pore. This could mean that the true structures form interactions that would make them differ from predictions based on the assumption of an isolated molecule. However, given this caveat, when scanned across the protein structure databank, our predictions found partial matches to a variety of proteins known to exist in complex multimeric assemblies.

In the absence of high-resolution structural information on any of these proteins, we believe our predicted models will be of use in building pseudo-atomic models of the T3SS which may provide mechanistic insights. With the rapid advances seen in electron cryo-microscopy, these models, together with stoichiometric data, may form the basis for interpreting medium resolution EM density maps to build pseudo-atomic models of the T3SS. In combination with genetic approaches to verify contacts, it may also be possible to discriminate between inter- and intra-molecular contacts (which are otherwise not distinguishable with covariance analysis), and ultimately to support or reject alternative arrangements of the proteins within the complex.

## Supporting Information

S1 File Supporting figures and text. The full set of results for each individual method can be found in this file along with some additional analysis of the final models described above.
(PDF)
