# Supplementary material for: Molecular Models for the Core Components of the Flagellar Type-III Secretion Complex
Source: PLoS One. 2016 Nov 17;11(11):e0164047. doi: 10.1371/journal.pone.0164047 (PMC5113899; doi:10.1371/journal.pone.0164047)
Supplement: S1 File — The full set of results for each individual method can be found in this file along with some additional analysis of the final models described above. (PDF) [file pone.0164047.s001.pdf]

Supporting Information

FlhA

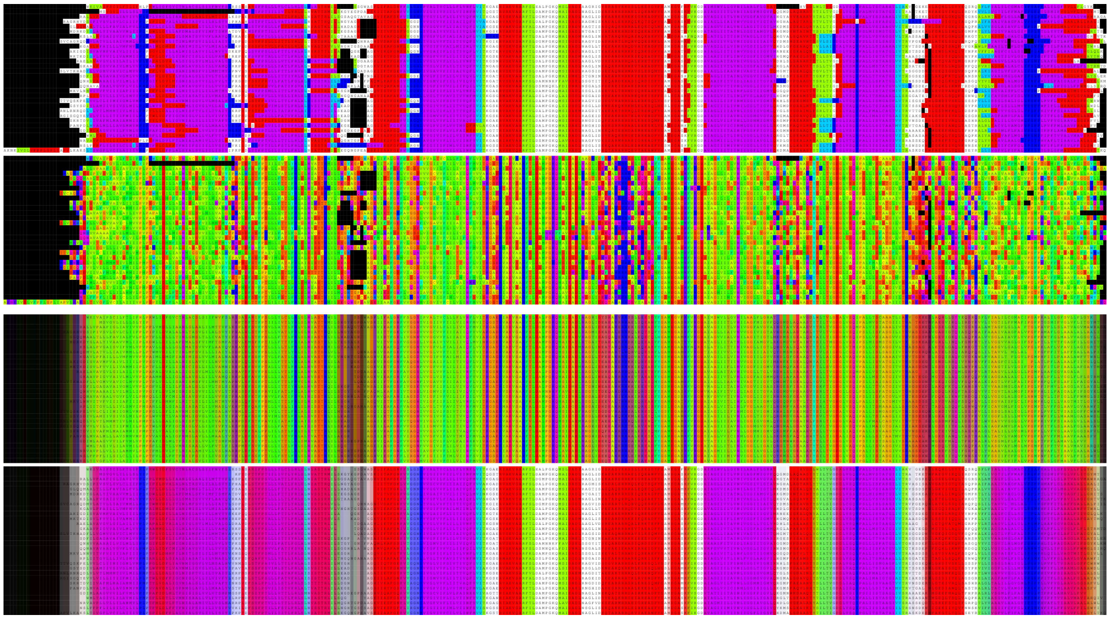

Fig S 1. FlhA Sequence alignment of 30 representative proteins. Each (identical) alignment block is coloured by predicted structure (top and bottom blocks) with the middle pair coloured by amino acid property (green = hydrophobic, red = negative, blue = positive charge or partial charge). The colours in the lower two blocks are averaged to emphasise conserved positions). Secondary structures are colour coded as red = alpha, green = beta which are overlayed with an added blue component to indicate predicted TM segments. As these typically overlay red α-helix predictions, the resulting purple hue can be taken to identify the TM-segments which are clearer in the averaged colours (lower panels)

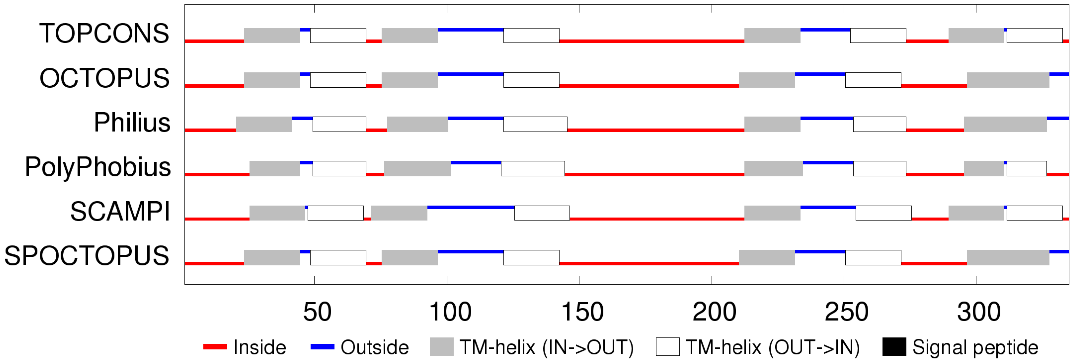

Fig S 2. FlhA membrane topology prediction. The summary of the TOPCONS predictions (identified by the name of the method to the right) was taken from the server with its explanatory colouring key. Inside (IN) in the context of the current proteins is the bacterial lumen (cytoplasm) and outside (OUT) is the periplasmic space.

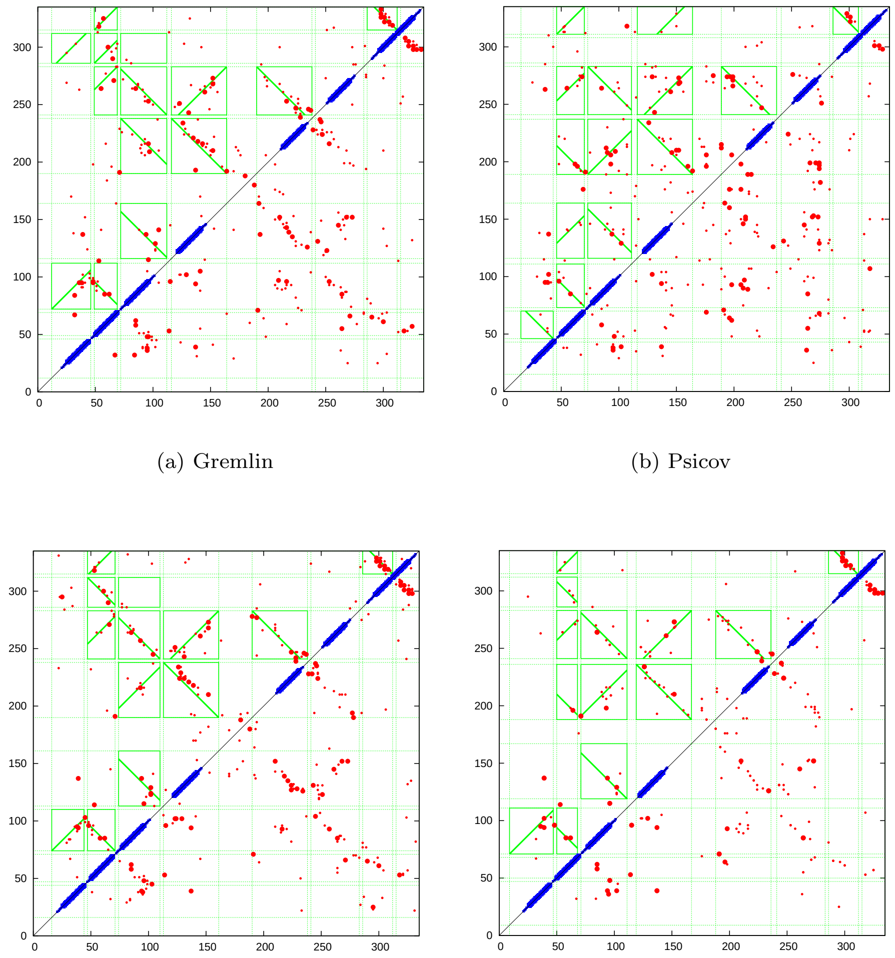

(a) Gremlin (b) Psicov

(c) EVfold (d) combined

Fig_S 3. Predicted contacts for FlhA Each set of predicted contacts (*a...d*) is plotted as a (symmetric) contact map showing the top scoring 300 contacts (red dots) with the top 50 shown as a slightly larger dot. (Contacts between positions closer that 5 were excluded.) The source of the data set is indicated below each matrix. The TOPCONS predition is plotted along the diagonal as a thick bar when all methods agree and a thin bar when two or more agree. The green boxes were calculated by the contact parsing algorithm outlined in the Methods section with a diagonal line indicating the preferred packing orientation. (Note: this always links two opposite edges of the box and do does not always coincide with the best regression fit used in its calculation).

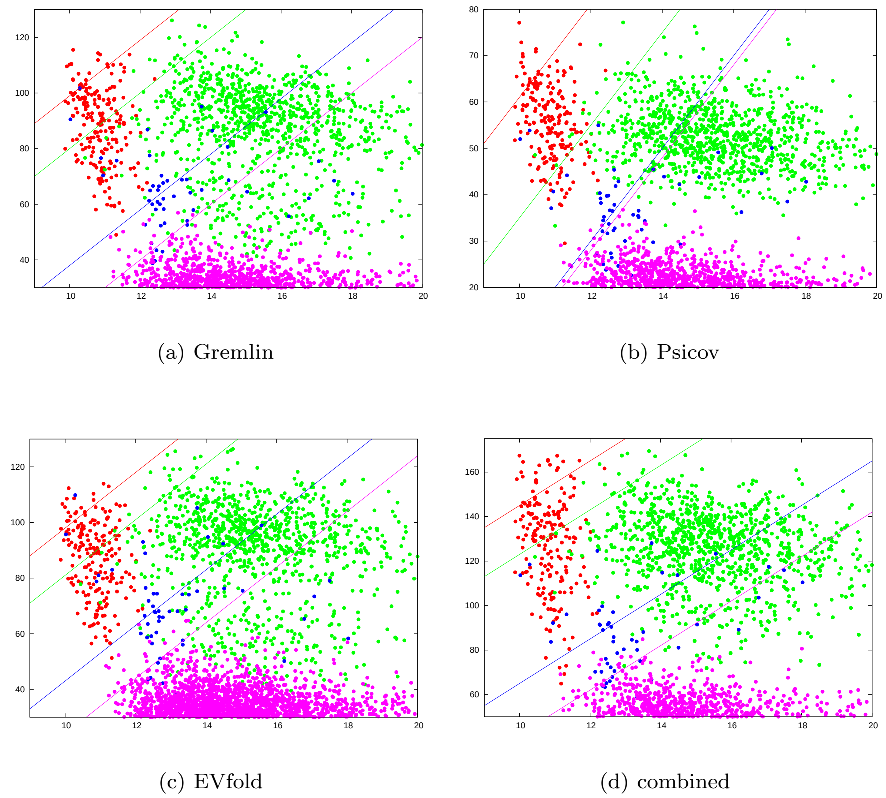

Fig_S 4. Score plots for the FlhA models The models generated by each of the four construction methods were scored with each set of predicted contacts (*a*...*d*) with each model represented by a dot plotted by its radius-of-gyration (X-axis) against score (Y-axis). The construction methods are colour coded as: red = TMpack, green = FILM3, blue = EVfold and Rosetta = magenta. The diagonal lines on each plot are similarly colour coded and mark the cutoff line below which (low-scoring, less-compact) models were discarded. The lines were adjusted to 'slice' off roughly equal numbers of models from each method/constraint combination.

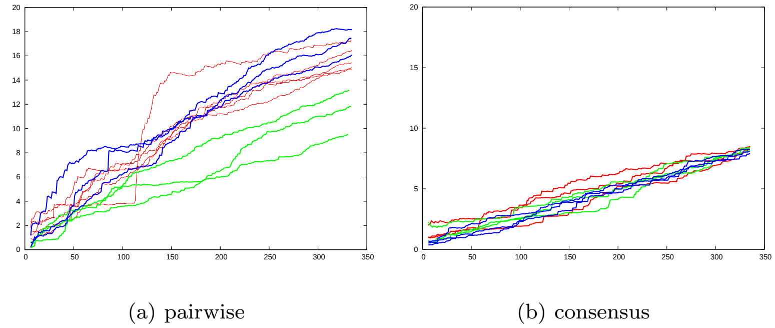

Fig_S 5. Comparison of the clustered models. *a*) The three FILM3 models at the heart of the fold-space cluster (F4, F6, F8) were compared with the TMpack model (T10) that has the same fold (blue traces) and other neighbouring TMpack models (red). For comparison the FILM3 models were compared to each other (green). *b*) The final consensus model constructed by Modeller was compared to the component models provided as input ("homology" models). These were the three FILM3 models (4,6,8) and three variants of the TMpack model (T10). Three variants of the model corresponding to the idealised packing model Fig 4 were also compared (blue). Parts *a* and *b* have been kept on the same RMSD scale (Y-axis) for ease of comparison with each other and subsequent plots. This value is the unweighted RMSD over increasingly larger sets of residues (Y-axis) as ranked by their residue-level `SAP` score.

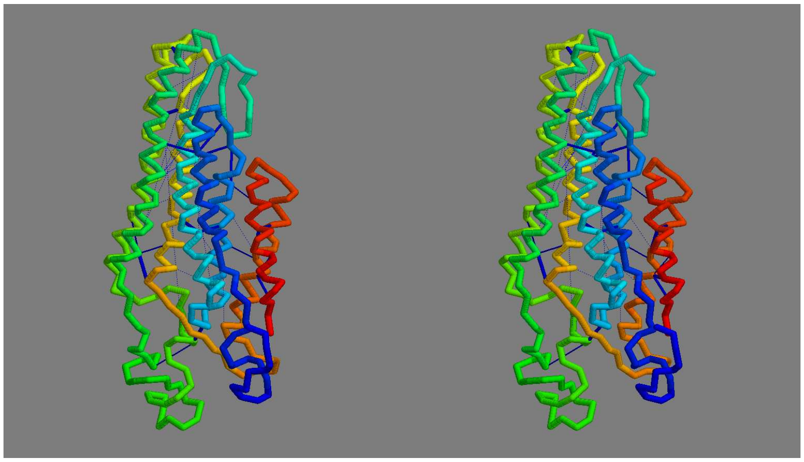

(a) consensus helices

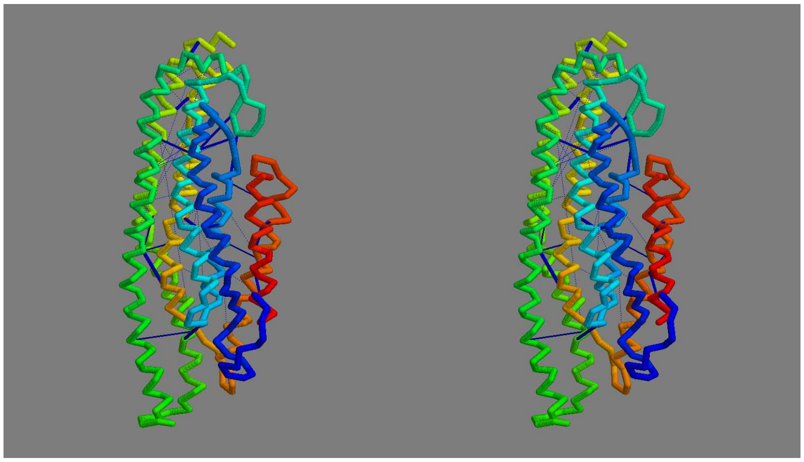

(b) extended helices

Fig_S 6. Final FlhA model. The final consensus model for FlhA is shown in frame *a* as a stereo pair, coloured from blue (amino) to red (carboxy terminus). The orientation is such that the cytoplasmic globular domain would lie (well) below. Part *b* shows the model constructed but with the helices extended into the regions suggested by the contact-parsing program. The strongest predicted contact pairs are linked by dark-blue bars.

FlhB

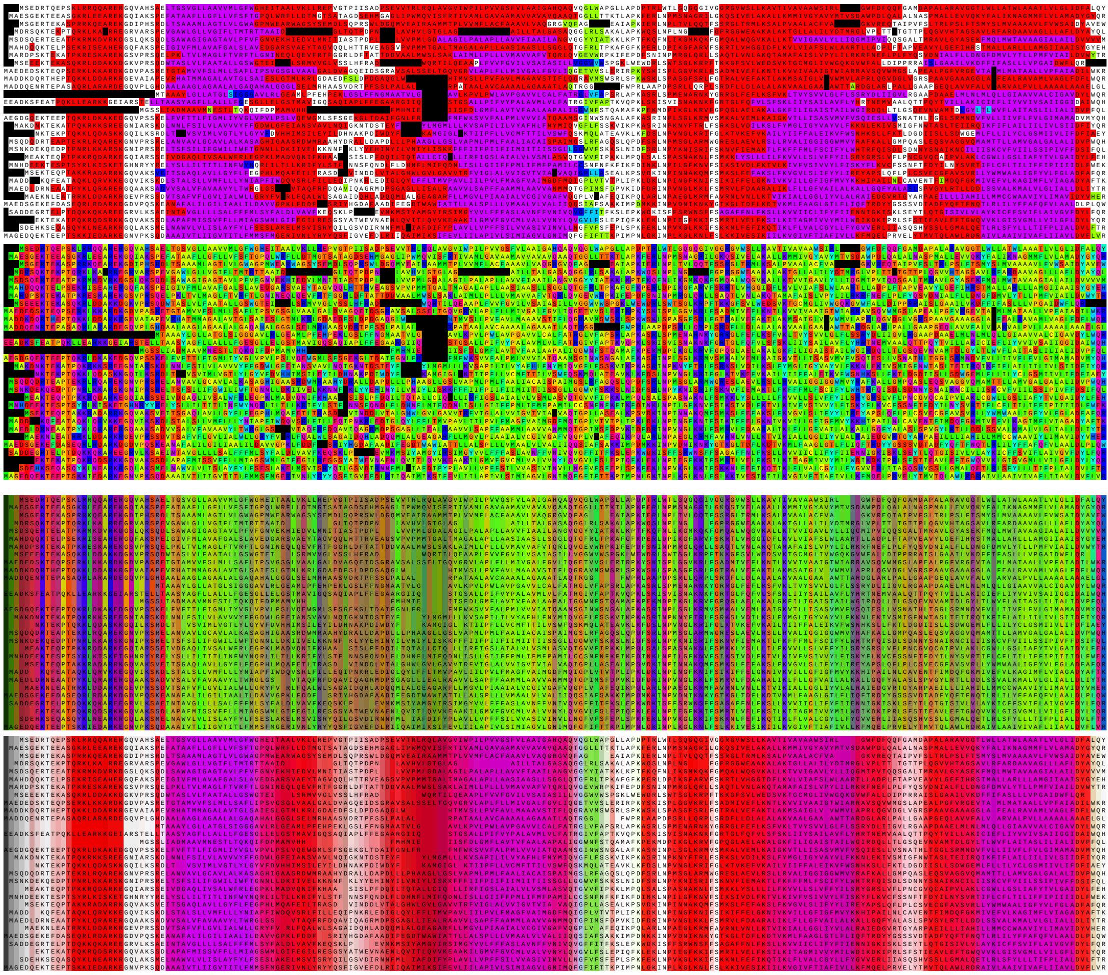

Fig_S 7. FlhB sequence alignment of 30 representative proteins. The double helical hairpin structure is apparent in the predicted secondary structures and the four MEMSAT predicted TM-segments overlay these helices. (See the legend to Fig_S1 for an explanation of the colouring scheme). The extra amino terminal helix can be seen to be very polar (red/blue colours in the lower panels) and the TM-segments are equally hydrophobic in nature (green). In these regions, there are very few conserved positions, with the exception of a conserved positive charge (K) and a conserved negative charge (D) at the termini of the second helical hairpin.

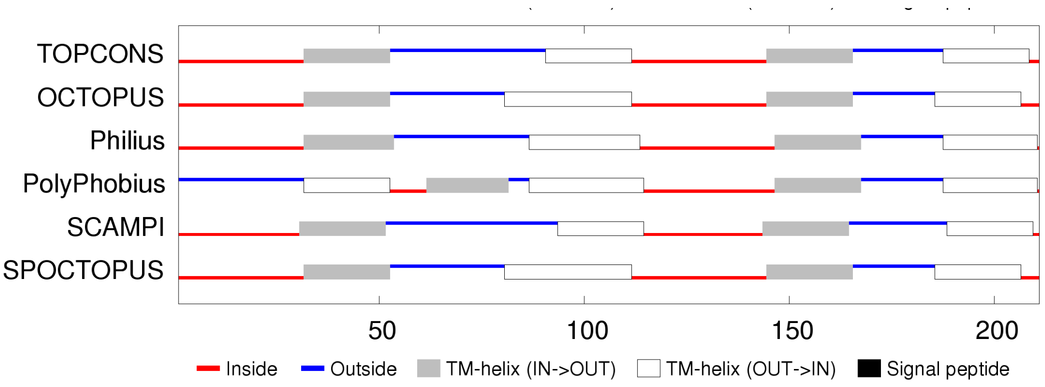

Fig_S 8. FlhB membrane topology prediction. Bar one, the methods employed in TOPCONS server are consistent in their predicted topologies.

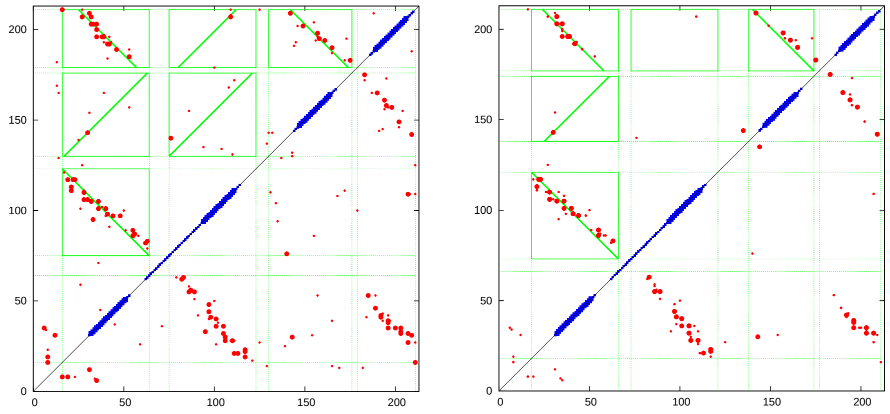

(a) Gremlin (b) combined

Fig_S 9. Predicted contacts for FlhB are plotted as in Fig_S3. As the predictions were consistent, only one example contact map is shown (*a*) along with the combined contacts from the three methods (*b*).

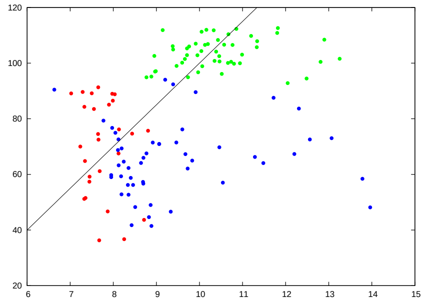

(a) Gremlin

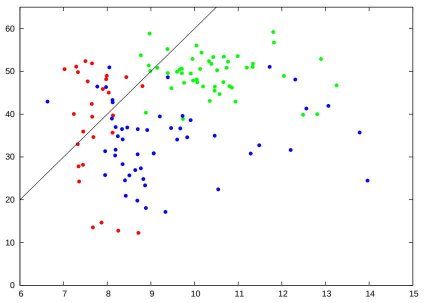

(b) Psicov

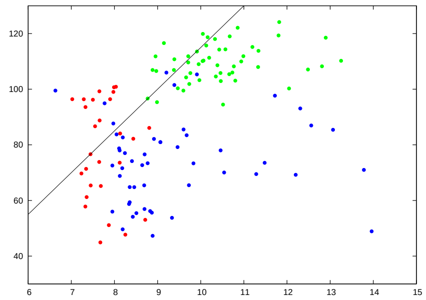

(c) EVfold

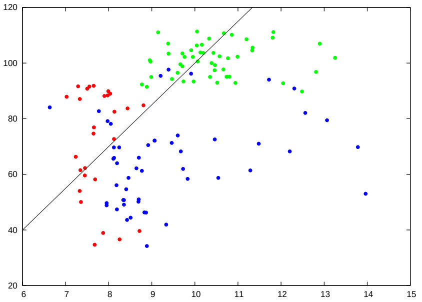

(d) combined

Fig_S 10. Score plots for the FlhB models. The models generated by each of the four construction methods were scored with each set of predicted contacts (*a*...*d*). (See legend to Fig_S4 for an explanation of the colours. As the methods all scored in a similar range, a single cutoff (black diagonal line) was used to discard poor models (lower, left of the line).

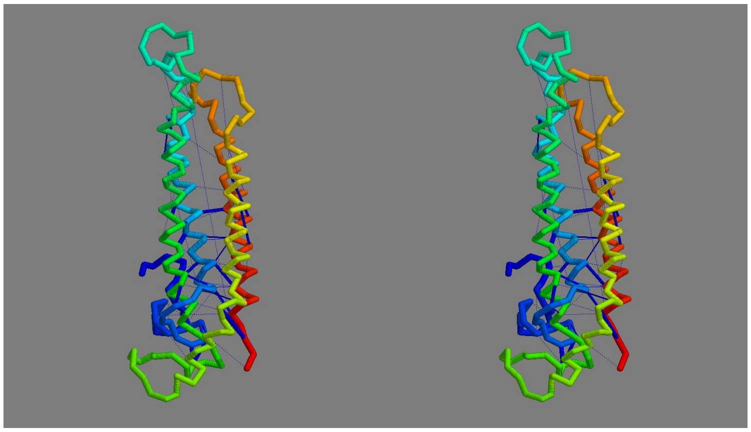

(a) Consensus model

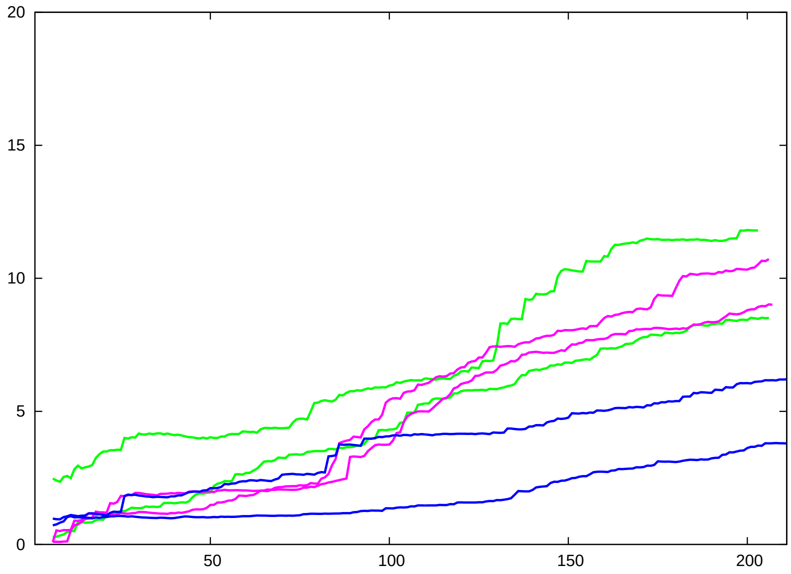

(b) RMSD to templates

Fig_S 11. Final FlhB model. The final consensus model for FlhB is shown in frame *a* as a stereo pair, coloured from blue (amino) to red (carboxy terminus). The interior of the cell would lie below the model. Part *b* shows the cumulative RMSD plots of the consensus model against the set of models from which it was constructed by Modeller.

FliP

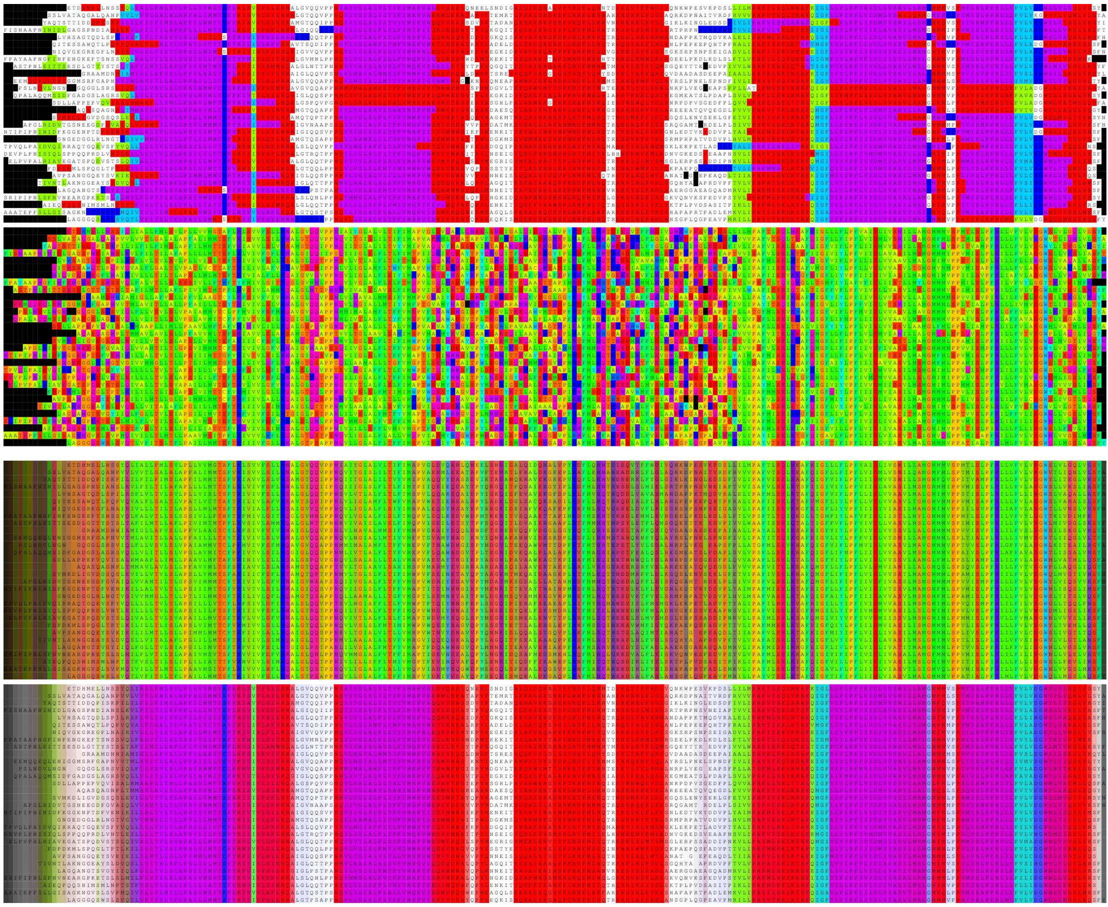

Fig_S 12. FliP sequence alignment of 30 representative proteins. (See the legend to Fig_S1 for an explanation of the colouring scheme).

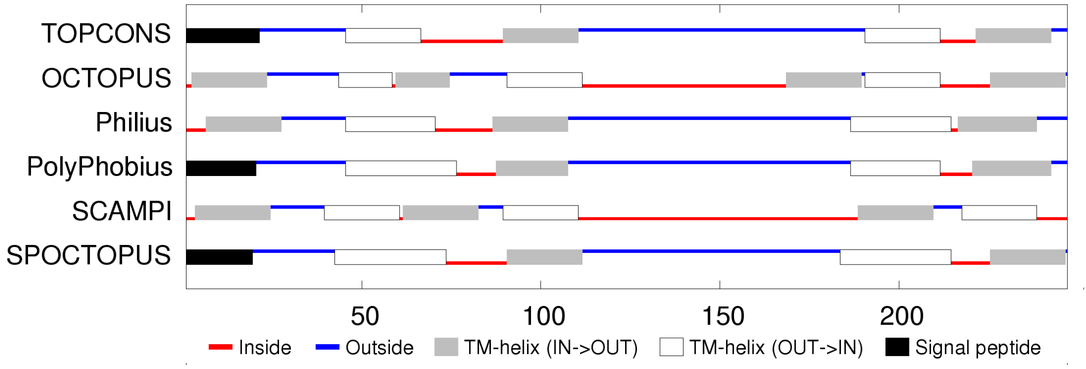

Fig_S 13. FliP membrane topology prediction.

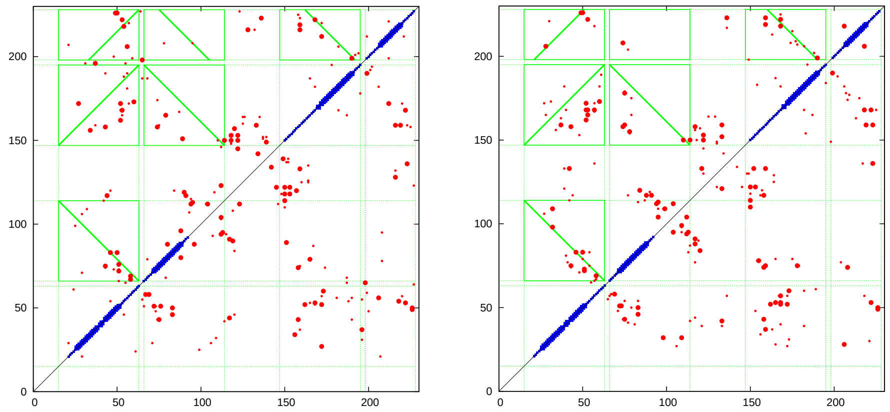

(a) Gremlin

(b) Psicov

Fig_S 14. Predicted contacts for FliP (4 helices) plotted as in Fig_S3.

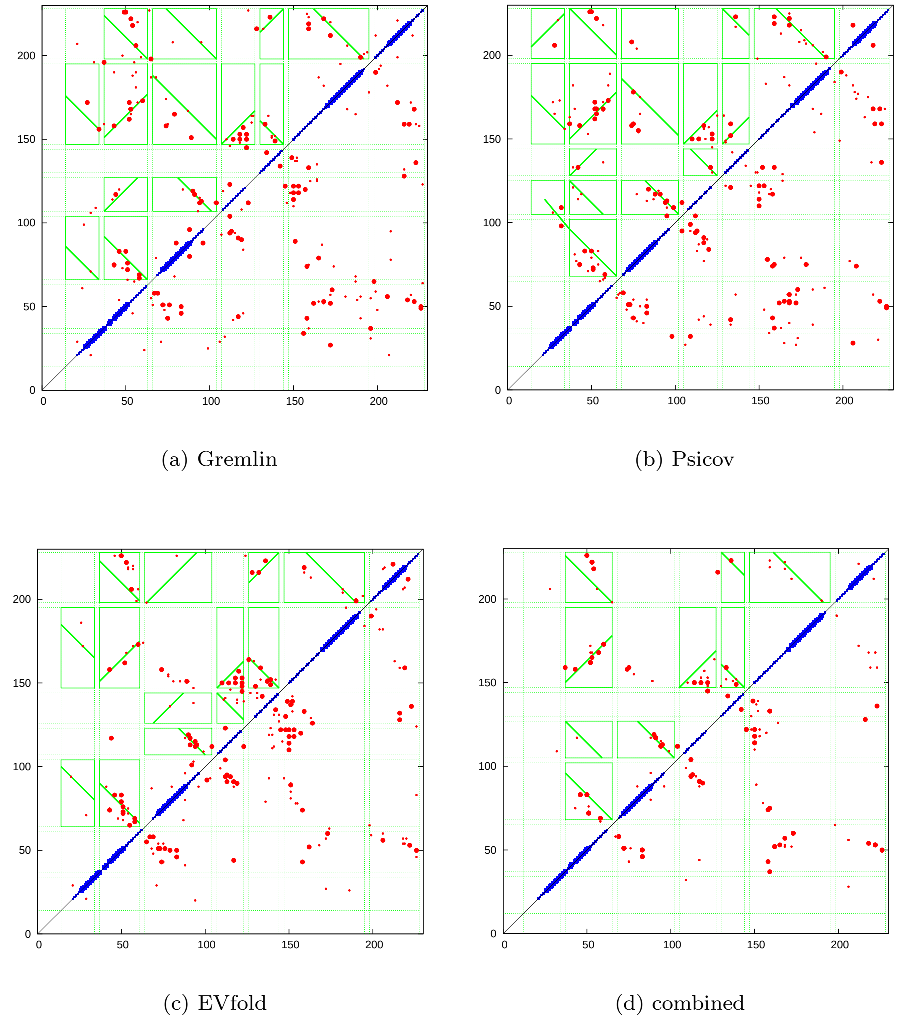

(a) Gremlin (b) Psicov

(c) EVfold (d) combined

Fig_S 15. Predicted contacts for fliP (7 helices) plotted as in Fig_S3.

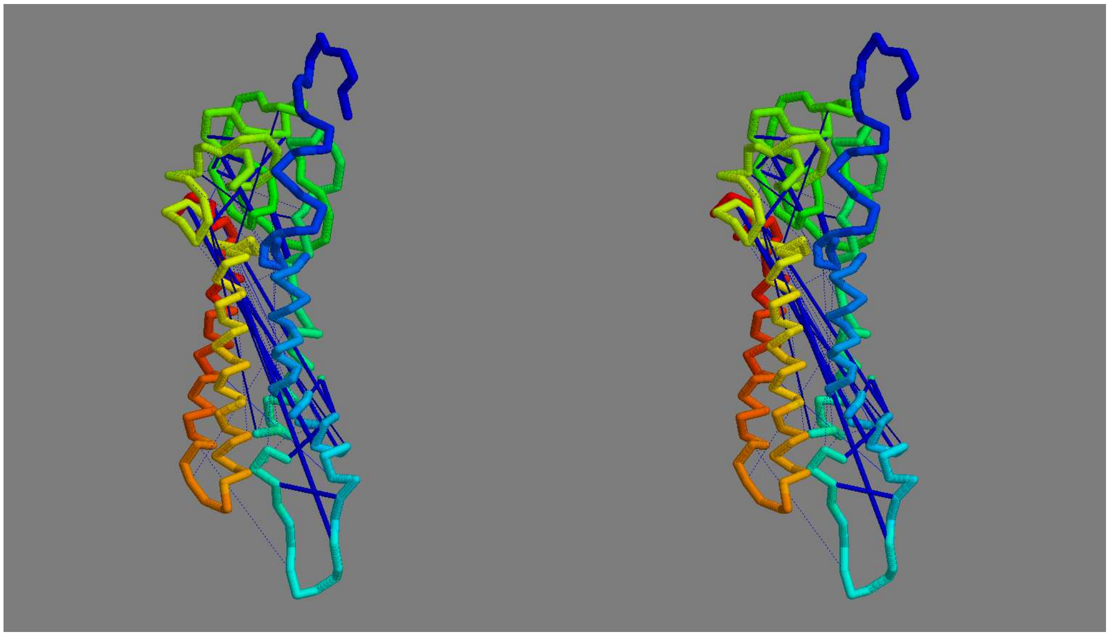

Fig_S 16. Four-helix bundle FliP model. Although the helix packing results in a compact structure, many predicted contacts are too long (dark blue rods). These could be improved by flipping the orientation of the terminal pair of helices (yellow–red) but this would place the carboxy terminus on the side of the membrane where predictions and experiments indicate it should not be found.

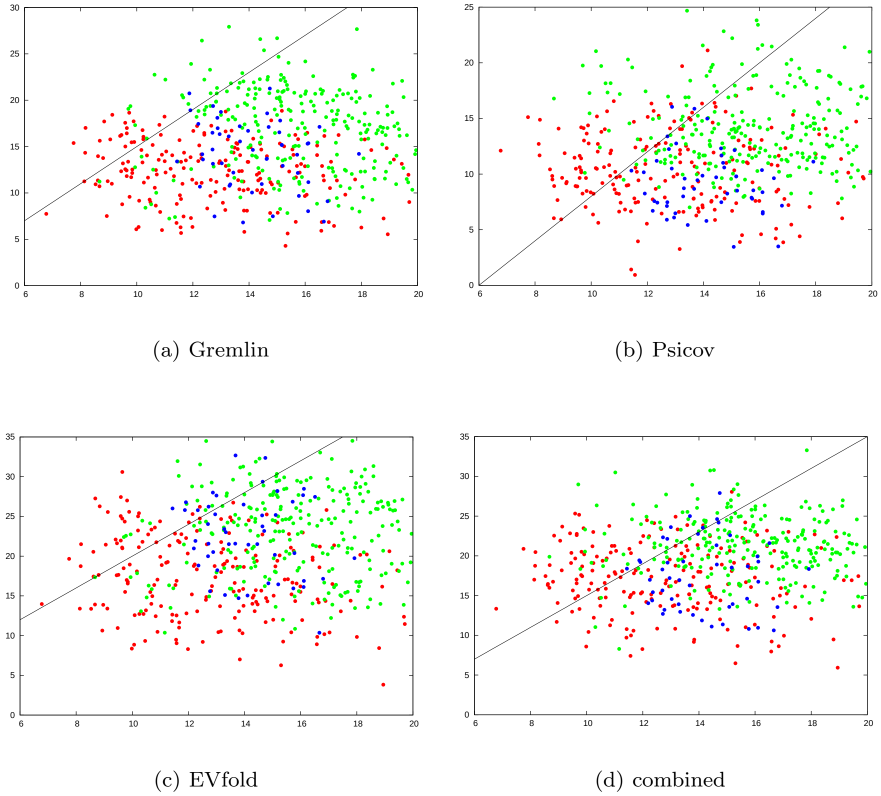

Fig_S 17. Score plots for the FliP models. The models generated by the four construction methods were scored with each set of predicted contacts (a...d). (See legend to Fig_S4). A single cutoff (black line) was used to discard poor models.

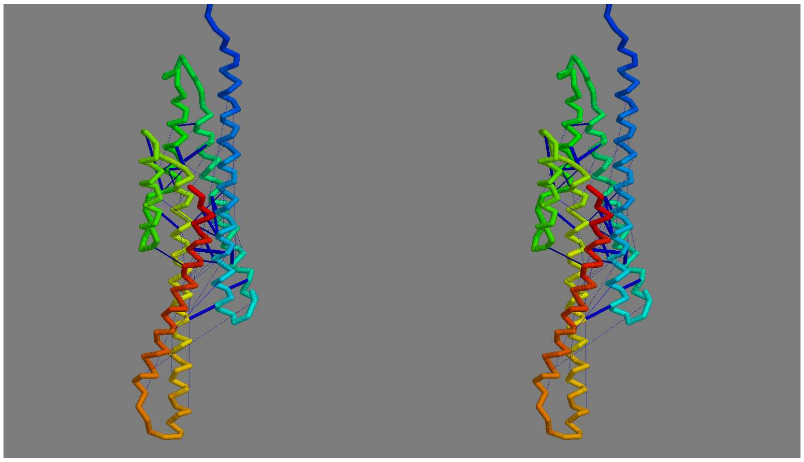

(a) Consensus model

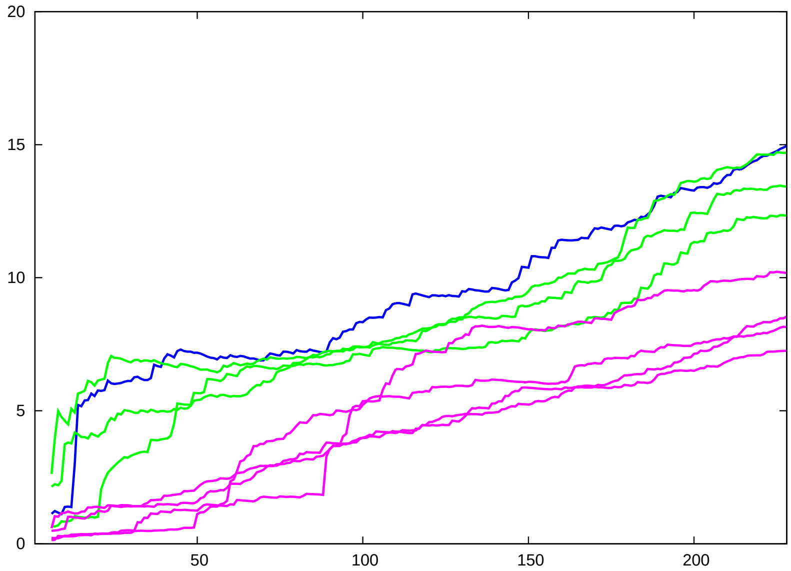

(b) RMSD to templates

Fig_S 18. Final FliP model. The final consensus model for FliP is shown in frame *a* as a stereo pair, coloured from blue (amino) to red (carboxy terminus). The interior of the cell would lie below the model. Compared to Fig_S16, the contacts (dark blue bars) are now all much shorter. Part *b* shows the cumulative RMSD plots of the consensus model against the set of models from which it was constructed.

FliQ

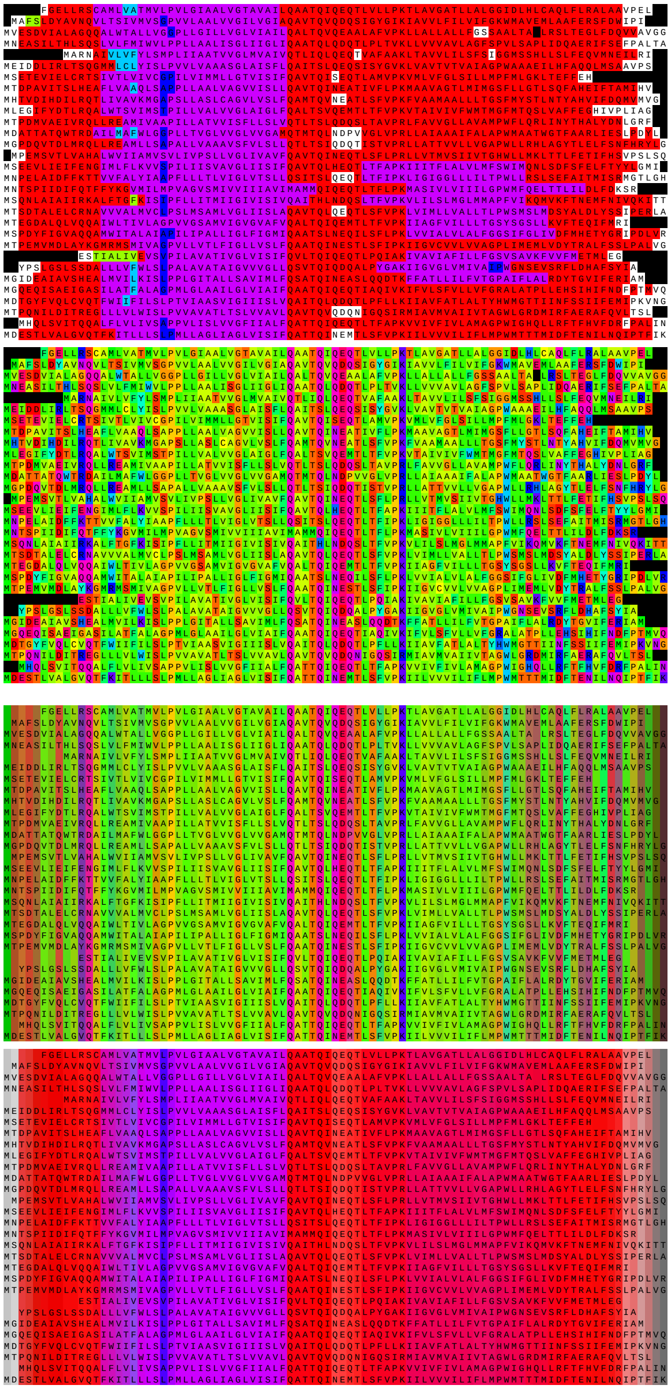

Fig_S 19. Sequence alignment with an added blue component to indicate predicted TM segments. As these typically overlay red α-helix predictions, the resulting purple hue can be taken to identify the TM-segments which are even clearer in the averaged colours (lower panels)

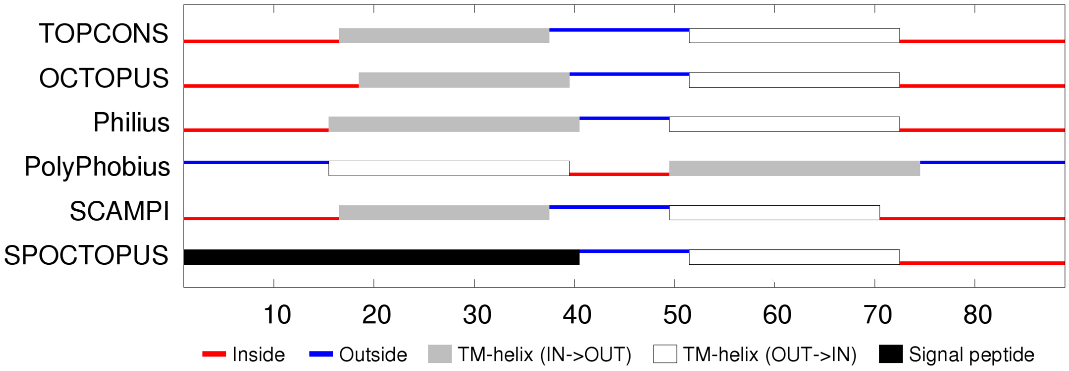

Fig_S 20. FliQ membrane topology prediction showing a consensus for cytoplasmic termini.

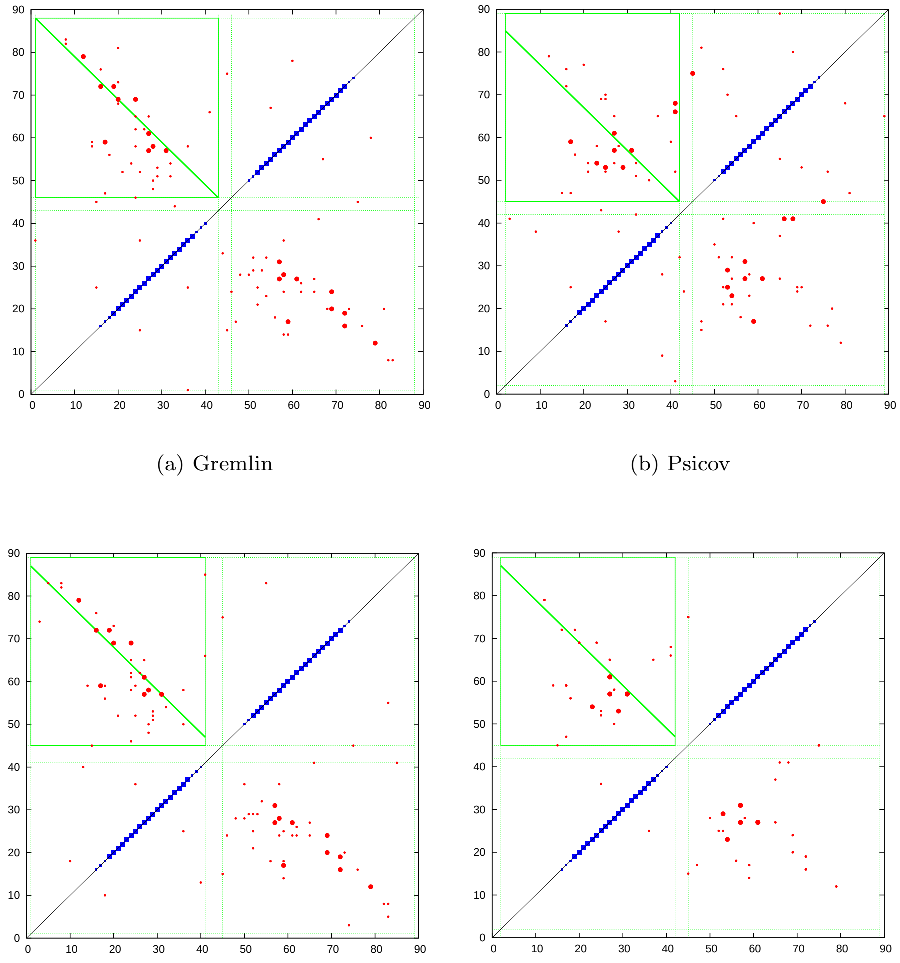

(a) Gremlin

(b) Psicov

(c) evfold

(d) consensus

Fig_S 21. Predicted contacts plotted as Fig_S3. Although simple, the methods show a consistent birfucation of the interaction.

FliR

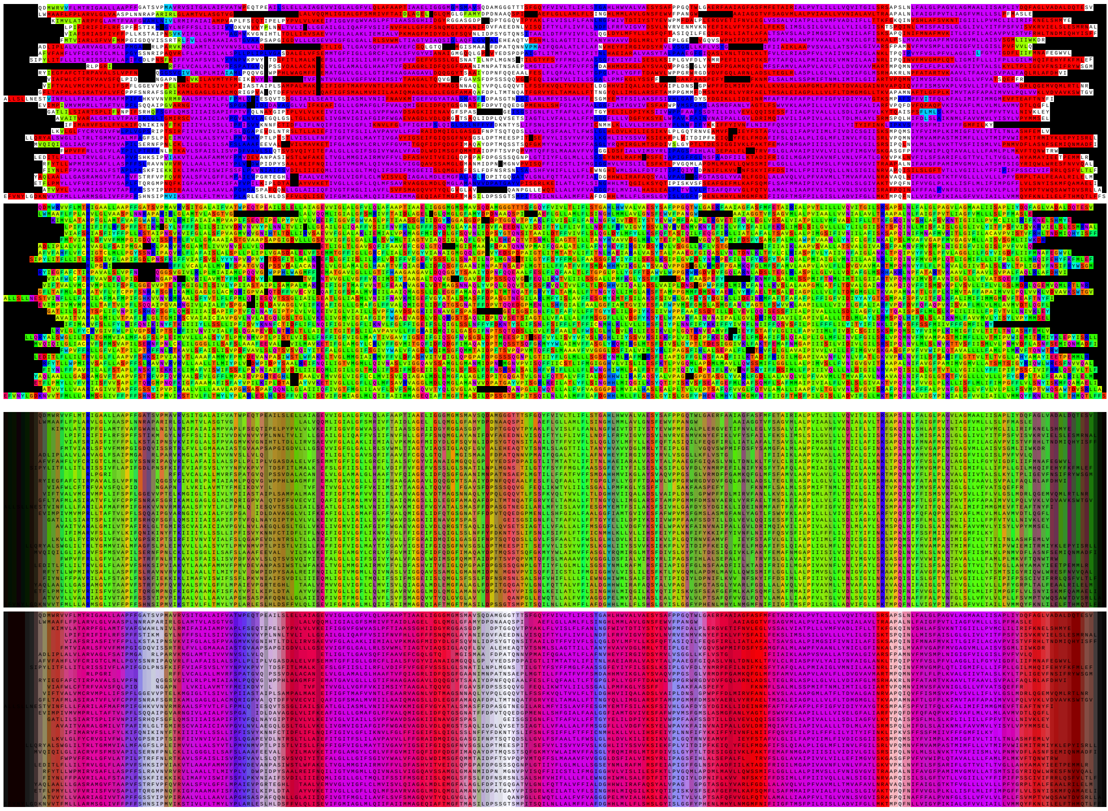

Fig_S 22. FliR sequence alignment of 30 representative proteins. (See the legend to Fig_S1 for an explanation).

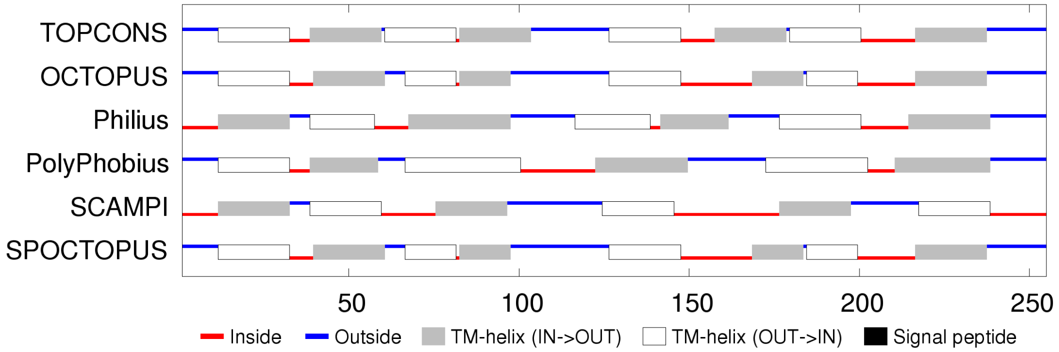

Fig_S 23. FliP membrane topology prediction.

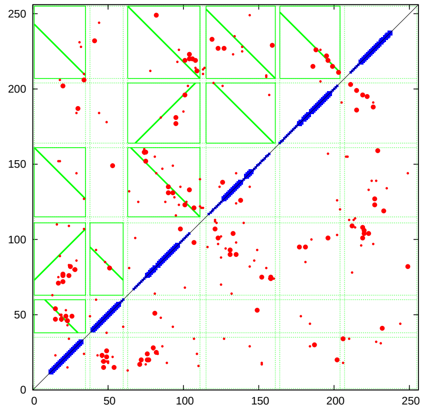

(a) Gremlin

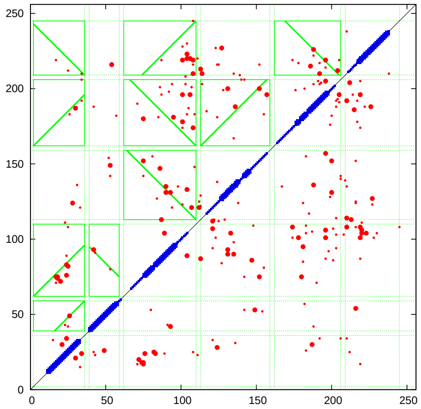

(b) Psicov

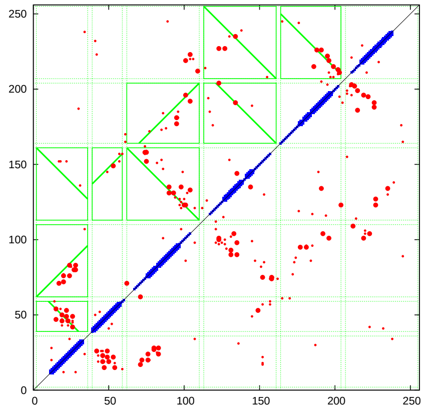

(c) EVfold

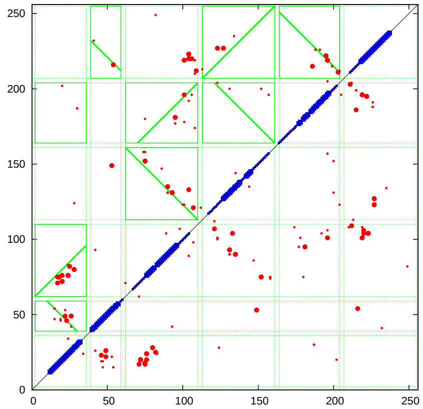

(d) combined

Fig_S 24. Predicted contacts for fliR plotted as in Fig_S3.

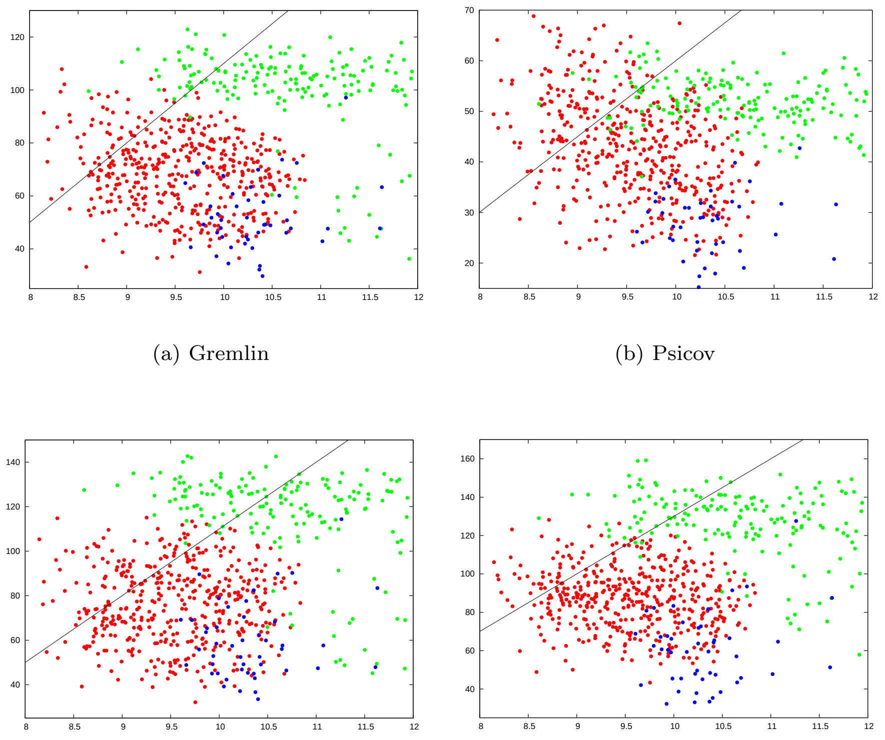

(a) Gremlin (b) Psicov

(c) EVfold (d) combined

Fig_S 25. Score plots for the FliR models. The models generated by the four construction methods were scored with each set of predicted contacts (*a*...*d*). (See legend to Fig_S4). A single cutoff (black line) was used to discard poor models. (This included all the EVfold models).

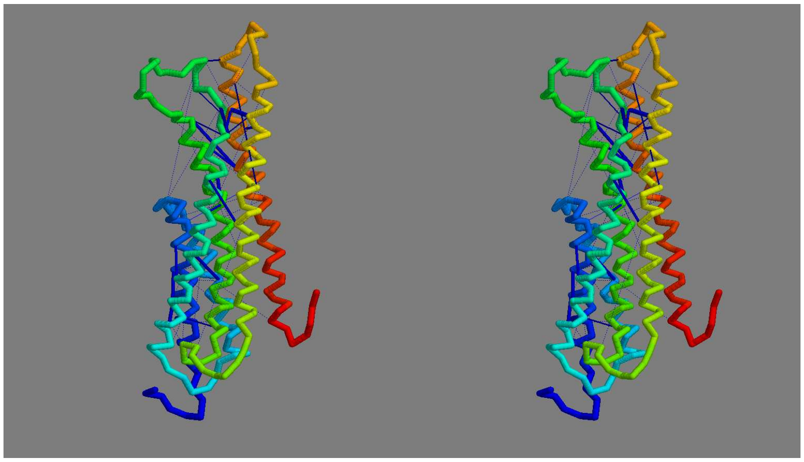

(a) Consensus model

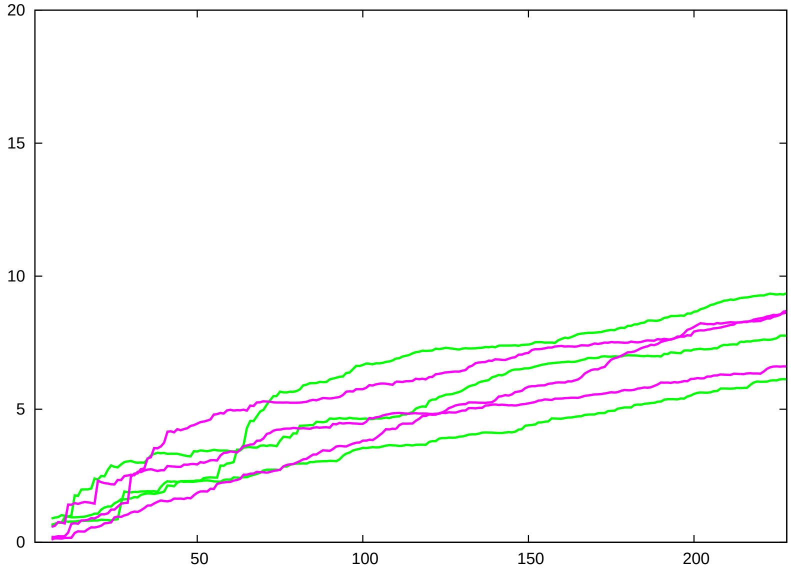

(b) RMSD to templates

Fig_S 26. Final FliR model. The final consensus model is shown in frame *a* as a stereo pair, coloured from blue (amino) to red (carboxy terminus). The interior of the cell would lie below the model. Part *b* shows the cumulative RMSD plots of the consensus model against the set of models from which it was constructed.

Comparisons between predicted models

The final predicted models for each protein all share the common characteristic of having a bundle of at least four long helices that appear to include extensions reaching beyond the normal confines of a lipid bilayer membrane. To see if there were any mode specific features shared between the models, each was compared to each other using the `SAP` program [51] (with, obviously, no constraint to force a one-to-one sequence match).

Core bundle topologies

There was little similarity between the FlhA core (involving helices 3...6) and either flhB or fliR (helices 3...6) nor was there any great similarity between the flhB and fliR cores. The reason for this became apparent on examination of the packing of each bundle. Viewed in a consistent orientation looking along the first helix at the top, designated North (N), then the packing of helices can be summarised by their progress around the cardinal points of the compass as: FlhA = NWSE, FlhB = NESW, FliR = NWES, FliP = NWSE. Only FlhA and FliP share a common core and had a good similarity of 5Å over 150 positions but as these proteins are very different in other ways, the correspondance is unlikely to be of any biological significance.

Internal similarities

Using the feature of `SAP` that allows identical proteins to be compared while avoiding the trivial one-to-one solution, it is possible to automatically investigate internal symmetry.

All the core bundles has some degree of internal two-fold symmetry reflecting their simple arrangement but this was most extensive in the flhB model, as was anticipated from the initial view of their predicted secondary structures (Fig 27(a)).

More unexpectedly, there was also an almost equally extensive repeat in the full FliR model involving a screw symmetry of 90° and 20Å placing helices 1, 2 and 4 over 3, 5 and 6, with each half forming a three-helix bundle (Fig 27(b)). These groupings and the large shift along the bundle axis correspond roughly to the two distinct sub-domains of predicted contacts described above and the displacement between them. Such relationships are not uncommon and are often the result of gene-duplication combined with a domain swap and can even generate knotted topologies.

Comparisons between predicted models and known structures

If the models predicted above have any correspondance to their real counterparts, it is not impossible that they may have some structural similarity to a protein of known structure that could not be detected with sequence data alone. To check this unlikely but important possibility, each of the model proteins were scanned against the protein structure databank (PDB) using the DALI search program: `http://ekhidna.biocenter.helsinki.fi/dali_server/start`.

The top scoring hits for each model (Fig 28 to Fig 31) had DALI Z-scores ranging from 4 to 8. This is an estimated significance value and the higher end of this range would be considered highly significant. However, most of these "significant" hits were simple helix bundles or long helical hairpins that can be expected to recur widely.

However, some of these matches were quite extensive, such as the 161 positions aligned between the FlhA model and a bacterial permease PDB:`3b60-A` covering most of the core, or some matches of over 170 positions covering much of the FliP model. While these longer matches have little specific relevance, they indicate that the

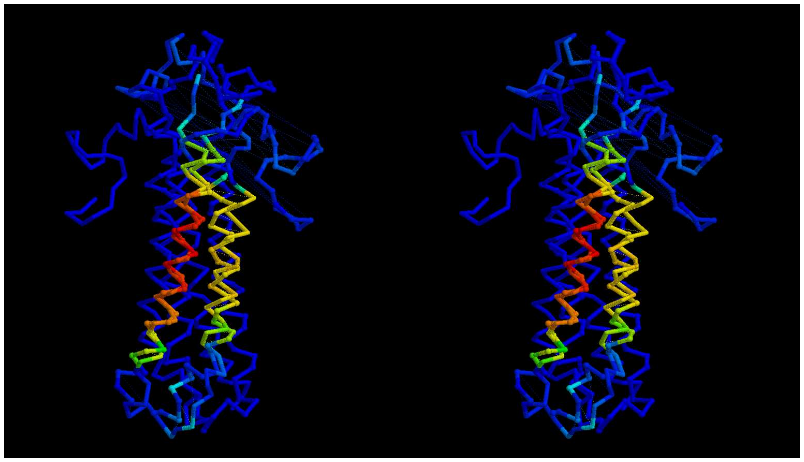

(a) FlhB

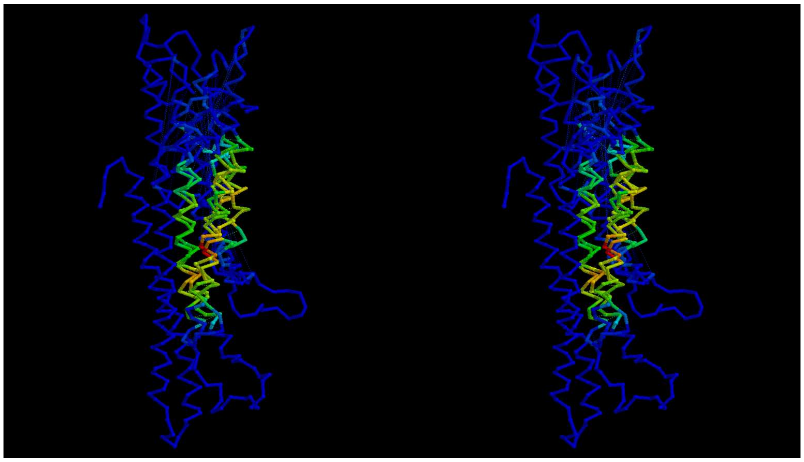

(b) fliR

Fig_S 27. Internal symmetries found by the SAP program included: *a* a two-fold relationship between the two helical hairpins in FlhB and *b* a helical screw relationship involving a triple of helices in FliR. In these stereo representations, both copies of the full (α-carbon) structures are superposed with the matching portions coloured by their degree of similarity (the SAP weight) running from red (high) to blue (low). One of the copies is distinguished by having small spheres on the α-carbon positions.

No:	Chain	Z	rmsd	lali	nres	%id	PDB	Description
1:	4e40-A	5.3	12.7	146	245	4	PDB	MOLECULE: PUTATIVE UNCHARACTERIZED PROTEIN;
2:	4tql-B	4.9	6.9	153	235	5	PDB	MOLECULE: THREE HELIX BUNDLE;
3:	1ya9-A	4.8	4.6	134	168	4	PDB	MOLECULE: APOLIPOPROTEIN E;
4:	3tul-A	4.8	6.5	123	136	6	PDB	MOLECULE: CELL INVASION PROTEIN SIPB;
5:	4uos-A	4.8	4.0	145	188	6	PDB	MOLECULE: DESIGNED HELICAL BUNDLE;
6:	2izp-A	4.6	5.1	150	272	4	PDB	MOLECULE: PUTATIVE MEMBRANE ANTIGEN;
7:	2yfb-B	4.6	6.6	151	238	7	PDB	MOLECULE: METHYL-ACCEPTING CHEMOTAXIS TRANSDUCER;
8:	3b60-A	4.5	6.0	161	572	4	PDB	MOLECULE: LIPID A EXPORT ATP-BINDING/PERMEASE PROTEIN MSBA;
9:	3caz-B	4.4	5.5	150	210	2	PDB	MOLECULE: BAR PROTEIN;
10:	3u0c-A	4.4	7.8	113	151	5	PDB	MOLECULE: INVASIN IPAB;
11:	2z0v-A	4.4	6.3	149	223	10	PDB	MOLECULE: SH3-CONTAINING GRB2-LIKE PROTEIN 3;
12:	4a3a-A	4.3	10.0	150	221	6	PDB	MOLECULE: AMPHIPHYSIN;
13:	1i49-A	4.2	6.6	137	201	4	PDB	MOLECULE: ARFAPTIN 2;
14:	1vct-A	4.2	4.2	115	193	10	PDB	MOLECULE: HYPOTHETICAL PROTEIN PH0236;
15:	1kmi-Z	4.2	11.4	129	177	3	PDB	MOLECULE: CHEMOTAXIS PROTEIN CHEY;
16:	1fio-A	4.2	9.4	149	190	7	PDB	MOLECULE: SSO1 PROTEIN;
17:	2b5u-A	4.2	7.9	119	470	9	PDB	MOLECULE: COLICIN E3;
18:	4wpc-B	4.1	6.0	139	288	6	PDB	MOLECULE: RHO GTPASE-ACTIVATING PROTEIN RGD1;
19:	4cv5-A	4.1	5.9	135	185	4	PDB	MOLECULE: GENERAL NEGATIVE REGULATOR OF TRANSCRIPTION SUBUN
20:	4igg-B	3.9	4.3	124	771	5	PDB	MOLECULE: CATENIN ALPHA-1;

Fig_S 28. FlhA top PDB matches. The columns specify the PDB identifer code and chain and an estimated Z-score along with the RMSD, alignment length (lali), protein length and percent sequence identity in the alignment.

No:	Chain	Z	rmsd	lali	nres	%id	PDB	Description
1:	4uos-A	8.1	4.6	150	188	8	PDB	MOLECULE: DESIGNED HELICAL BUNDLE;
2:	1ya9-A	7.0	5.5	134	168	4	PDB	MOLECULE: APOLIPOPROTEIN E;
3:	2yfb-B	6.8	5.3	116	238	9	PDB	MOLECULE: METHYL-ACCEPTING CHEMOTAXIS TRANSDUCER;
4:	1c17-M	6.6	3.6	122	142	14	PDB	MOLECULE: ATP SYNTHASE SUBUNIT C;
5:	3dyj-A	6.5	4.9	121	317	11	PDB	MOLECULE: TALIN-1;
6:	4xev-A	6.2	4.5	119	148	11	PDB	MOLECULE: FUSION PROTEIN OF PROTEIN-TYROSINE KINASE 2-BETA
7:	1k05-B	6.0	4.8	121	142	9	PDB	MOLECULE: FOCAL ADHESION KINASE 1;
8:	4k0d-B	5.9	5.7	114	145	8	PDB	MOLECULE: PERIPLASMIC SENSOR HYBRID HISTIDINE KINASE;
9:	3fyq-A	5.8	5.3	122	179	6	PDB	MOLECULE: CG6831-PA (TALIN);
10:	2j68-A	5.8	9.7	140	680	4	PDB	MOLECULE: BACTERIAL DYNAMIN-LIKE PROTEIN;
11:	2asr-A	5.8	6.3	123	142	8	PDB	MOLECULE: ASPARTATE RECEPTOR;
12:	1st6-A	5.7	5.5	115	1049	5	PDB	MOLECULE: VINCULIN;
13:	2m64-A	5.6	4.2	113	231	9	PDB	MOLECULE: PHLP5;
14:	2a01-A	5.6	5.2	132	243	7	PDB	MOLECULE: APOLIPOPROTEIN A-I;
15:	1y4c-A	5.5	4.4	111	480	3	PDB	MOLECULE: MALTOSE BINDING PROTEIN FUSED WITH DESIGNED
16:	4f4c-A	5.5	5.2	149	1250	4	PDB	MOLECULE: MULTIDRUG RESISTANCE PROTEIN PGP-1;
17:	4p9t-A	5.5	5.4	115	231	10	PDB	MOLECULE: CATENIN ALPHA-2;
18:	4m1m-A	5.4	5.0	154	1188	6	PDB	MOLECULE: MULTIDRUG RESISTANCE PROTEIN 1A;
19:	1lih-A	5.4	6.8	128	160	5	PDB	MOLECULE: ASPARTATE RECEPTOR;
20:	1uru-A	5.4	5.4	110	217	5	PDB	MOLECULE: AMPHIPHYSIN;
21:	4whj-A	5.3	4.9	143	565	8	PDB	MOLECULE: INTERFERON-INDUCED GTP-BINDING PROTEIN MX2;
22:	1o5h-A	5.3	11.3	144	200	10	PDB	MOLECULE: FORMIMINOTETRAHYDROFOLATE CYCLODEAMINASE;
23:	1szi-A	5.3	7.1	122	194	9	PDB	MOLECULE: MANNOSE-6-PHOSPHATE RECEPTOR BINDING PROTEIN 1;
24:	3ay5-A	5.3	4.1	105	298	5	PDB	MOLECULE: CYCLIN-D1-BINDING PROTEIN 1;

Fig_S 29. FlhB top PDB matches. (See previous legend).

No:	Chain	Z	rmsd	lali	nres	%id	PDB	Description
1:	2j68-A	5.9	7.2	148	680	5	PDB	MOLECULE: BACTERIAL DYNAMIN-LIKE PROTEIN;
2:	1s94-B	5.6	7.8	114	118	6	PDB	MOLECULE: S-SYNTAXIN;
3:	2xhe-B	5.5	11.7	151	220	3	PDB	MOLECULE: UNC18;
4:	4ntj-A	5.5	5.3	130	369	8	PDB	MOLECULE: P2Y PURINOCEPTOR 12, SOLUBLE CYTOCHROME B562;
5:	4k5y-A	5.4	4.0	113	407	10	PDB	MOLECULE: CORTICOTROPIN-RELEASING FACTOR RECEPTOR 1, T4-LYS
6:	1fio-A	5.4	7.9	134	190	4	PDB	MOLECULE: SSO1 PROTEIN;
7:	3s84-B	5.3	6.9	138	241	5	PDB	MOLECULE: APOLIPOPROTEIN A-IV;
8:	4ons-C	5.3	3.8	125	205	8	PDB	MOLECULE: CATENIN ALPHA-2;
9:	3oce-A	5.2	10.2	163	461	7	PDB	MOLECULE: FUMARATE LYASE:DELTA CRYSTALLIN;
10:	4wid-A	5.2	9.0	165	353	8	PDB	MOLECULE: RHUL123;
11:	4hgv-A	5.1	11.3	174	456	7	PDB	MOLECULE: FUMARATE HYDRATASE CLASS II;
12:	4eiy-A	5.1	6.7	158	390	9	PDB	MOLECULE: ADENOSINE RECEPTOR A2A/SOLUBLE CYTOCHROME B562 CH
13:	1jsw-B	5.1	12.1	177	460	7	PDB	MOLECULE: L-ASPARTATE AMMONIA-LYASE;
14:	3r6q-F	5.1	10.6	164	463	4	PDB	MOLECULE: ASPARTASE;
15:	4kik-B	5.1	7.8	139	650	6	PDB	MOLECULE: INHIBITOR OF NUCLEAR FACTOR KAPPA-B KINASE SUBUNI
16:	4kik-A	5.1	6.1	127	618	7	PDB	MOLECULE: INHIBITOR OF NUCLEAR FACTOR KAPPA-B KINASE SUBUNI
17:	3c98-B	5.1	14.2	144	230	6	PDB	MOLECULE: SYNTAXIN-BINDING PROTEIN 1;
18:	4k5y-C	5.1	4.4	117	248	9	PDB	MOLECULE: CORTICOTROPIN-RELEASING FACTOR RECEPTOR 1, T4-LYS
19:	4uy3-A	5.1	6.2	139	194	4	PDB	MOLECULE: SEPTATION RING FORMATION REGULATOR EZRA;
20:	3rrp-A	5.0	12.3	167	465	6	PDB	MOLECULE: PROBABLE FUMARATE HYDRATASE FUM;

Fig_S 30. FliP top PDB matches. (See previous legend).

```
No:  Chain   Z   rmsd lali  nres  %id PDB  Description
 1:  4u5a-B  6.9  4.4  134   184   10 PDB  MOLECULE: SPOROZOITE MICRONEME PROTEIN ESSENTIAL FOR CELL
 2:  4bem-J  6.5  9.2  152   182    8 PDB  MOLECULE: F1FO ATPASE C2 SUBUNIT;
 3:  1fio-A  6.4  9.3  155   190    6 PDB  MOLECULE: SSO1 PROTEIN;
 4:  1avo-B  6.4  6.8  124   140    3 PDB  MOLECULE: 11S REGULATOR;
 5:  2bl2-A  6.1  4.2  121   156   12 PDB  MOLECULE: V-TYPE SODIUM ATP SYNTHASE SUBUNIT K;
 6:  3lhp-S  6.0  8.6  111   116    6 PDB  MOLECULE: FV 4E10 HEAVY CHAIN;
 7:  2xhe-B  5.8  9.3  160   220    6 PDB  MOLECULE: UNC18;
 8:  2jsw-A  5.8  6.9  138   189    7 PDB  MOLECULE: TALIN-1;
 9:  3j9t-a  5.8  4.9  133   150    8 PDB  MOLECULE: V-TYPE PROTON ATPASE SUBUNIT D;
10:  4oyd-B  5.8  8.9  110   117   10 PDB  MOLECULE: APOPTOSIS REGULATOR BHRF1;
11:  5cwo-A  5.6  4.9  107   219    4 PDB  MOLECULE: DESIGNED HELICAL REPEAT PROTEIN;
12:  1fnt-c  5.6  8.3  140   198    7 PDB  MOLECULE: PROTEASOME COMPONENT C7-ALPHA;
13:  3s84-B  5.6  4.1  134   241    8 PDB  MOLECULE: APOLIPOPROTEIN A-IV;
14:  3lf9-A  5.6  9.1  113   120    7 PDB  MOLECULE: 4E10_D0_1IS1A_001_C (T161);
15:  1eh1-A  5.4  9.2  108   185    5 PDB  MOLECULE: RIBOSOME RECYCLING FACTOR;
16:  2d1l-A  5.3  5.6  131   249    5 PDB  MOLECULE: METASTASIS SUPPRESSOR PROTEIN 1;
17:  3l39-A  5.3  9.7  141   209    8 PDB  MOLECULE: PUTATIVE PHOU-LIKE PHOSPHATE REGULATORY PROTEIN;
18:  4x0r-A  5.3  5.2  143   201    7 PDB  MOLECULE: INTERFERON-INDUCED GTP-BINDING PROTEIN MX2;
19:  4r61-A  5.3  9.5  123   140    6 PDB  MOLECULE: GP41-BASED CONSTRUCT COVNHR3-ABC;
20:  1is1-A  5.3  8.9  113   185    6 PDB  MOLECULE: RIBOSOME RECYCLING FACTOR;
```

Fig_S 31. FliR top PDB matches. (See previous legend).

predicted models fall within the range of known structures some of which are occupying a similar integral membrane or pore-forming environment — such as the translocator protein bipD (PDB:`3nft`) from the type-III secretion system. Despite the context and being the third top hit to our FlhA model, this protein was topologically quite distinct from any of our predictions.
